# Awake prone position reduces work of breathing in patients with COVID-19 ARDS supported by CPAP

**DOI:** 10.1186/s13613-021-00967-6

**Published:** 2021-12-20

**Authors:** Davide Chiumello, Elena Chiodaroli, Silvia Coppola, Simone Cappio Borlino, Claudia Granata, Matteo Pitimada, Pedro David Wendel Garcia

**Affiliations:** 1 Department of Anesthesia and Intensive Care, ASST Santi Paolo E Carlo, San Paolo University Hospital, Via A. di Rudinì 8, Milan, Italy; 2 Department of Health Sciences, University of Milan, Milan, Italy; 3 Coordinated Research Center On Respiratory Failure, University of Milan, Milan, Italy; 4 Institute of Intensive Care Medicine, University Hospital of Zurich, Zurich, Switzerland

**Keywords:** Continuous positive airway pressure, Awake prone position, Helmet CPAP, ARDS, COVID-19, Work of breathing

## Abstract

**Background:**

The use of awake prone position concomitant to non-invasive mechanical ventilation in acute respiratory distress syndrome (ARDS) secondary to COVID-19 has shown to improve gas exchange, whereas its effect on the work of breathing remain unclear. The objective of this study was to evaluate the effects of awake prone position during helmet continuous positive airway pressure (CPAP) ventilation on inspiratory effort, gas exchange and comfort of breathing.

**Methods:**

Forty consecutive patients presenting with ARDS due to COVID-19 were prospectively enrolled. Gas exchange, esophageal pressure swing (ΔPes), dynamic transpulmonary pressure (dTPP), modified pressure time product (mPTP), work of breathing (WOB) and comfort of breathing, were recorded on supine position and after 3 h on prone position.

**Results:**

The median applied PEEP with helmet CPAP was 10 [8–10] cmH_2_O. The PaO_2_/FiO_2_ was higher in prone compared to supine position (Supine: 166 [136–224] mmHg, Prone: 314 [232–398] mmHg, *p* < 0.001). Respiratory rate and minute ventilation decreased from supine to prone position from 20 [17–24] to 17 [15–19] b/min (*p* < 0.001) and from 8.6 [7.3–10.6] to 7.7 [6.6–8.6] L/min (*p* < 0.001), respectively. Prone position did not reduce ΔPes (Supine: − 7 [− 9 to − 5] cmH_2_O, Prone: − 6 [− 9 to − 5] cmH_2_O, *p* = 0.31) and dTPP (Supine: 17 [14–19] cmH_2_O, Prone: 16 [14–18] cmH_2_O, *p* = 0.34). Conversely, mPTP and WOB decreased from 152 [104–197] to 118 [90–150] cmH_2_O/min (*p* < 0.001) and from 146 [120–185] to 114 [95–151] cmH_2_O L/min (*p* < 0.001), respectively. Twenty-six (65%) patients experienced a reduction in WOB of more than 10%. The overall sensation of dyspnea was lower in prone position (*p* = 0.005).

**Conclusions:**

Awake prone position with helmet CPAP enables a reduction in the work of breathing and an improvement in oxygenation in COVID-19-associated ARDS.

**Supplementary Information:**

The online version contains supplementary material available at 10.1186/s13613-021-00967-6.

## Background

The SARS-CoV-2 infection can result in coronavirus 2019 disease (COVID-19). The clinical spectrum of COVID-19 can range from mild to critical forms of acute respiratory distress syndrome (CARDS, COVID-19-associated ARDS) with an associated in-hospital mortality of up to 84% [1, 2]. Non-invasive and invasive mechanical ventilation have been reportedly employed in up to 62 and 36%, respectively, of the critically ill COVID-19 patients requiring respiratory support [2–4]. Similarly to classic ARDS, the clinical management of CARDS is intended to provide a lung protective strategy enabling adequate gas exchange and limiting damage to the lung (i.e., ventilation induced lung injury (VILI)) [5].

In the early phase of the pandemic, based on previous, positive studies on the use of prone position in ARDS [6, 7], international scientific societies recommended the use of prone position in moderate to severe mechanically ventilated CARDS [8, 9]. The use of prone position in mechanically ventilated CARDS patients significantly improved the oxygenation without any major changes in lung compliance [10, 11]. Recently, prone position has been employed in non-invasively mechanically ventilated CARDS patients (“awake prone position”) to improve oxygenation [12–15]. However, in the absence of data regarding its effect on endotracheal intubation rate and mortality, no firm recommendations have been issued on the use of awake prone position [8, 16–18].

In addition to severe hypoxemia, CARDS suffering patients are characterized by an increased respiratory rate and inspiratory effort that in turn can promote patient self-inflicted lung injury (PSILI) [19]. Non-invasive respiratory support, especially in the form of helmet CPAP, has been suggested to reduce the inspiratory effort and possibly, the requirement of invasive mechanical ventilation [20–22]. Awake prone position, employed synergistically with non-invasive respiratory support, has been hypothesized to result in a decrease of inspiratory effort, dyspnea and lower rates of intubation, by promoting better lung inflation and recruitment [12, 15, 17, 23].

In light of the scarcity of data regarding the benefits of awake prone position concomitant to non-invasive respiratory support in CARDS, we aimed to evaluate the effect of awake prone position in helmet CPAP ventilated patients by comparing it to supine position regarding the reduction of inspiratory effort, the improvement in gas exchange and comfort of breathing.

## Methods

### Study population

All adults (> 18 years), presenting with a laboratory-confirmed SARS-CoV-2 infection and suffering acute hypoxemic respiratory failure with a PaO_2_/FiO_2_ ratio < 300 mmHg under application of a PEEP ≥ 5cmH_2_O by means of helmet CPAP, displaying bilateral ground glass opacities on chest X-ray or computed tomography (CT) of the lung, admitted to the high dependency unit of the ASST Santi Paolo e Carlo Hospital, Milan, Italy from February to April 2021 were enrolled. Patients were excluded in case they required immediate endotracheal intubation, presented unstable hemodynamics or had a Glasgow Coma Scale < 15.

The study was approved by the institutional review board of the ASST Santi Paolo e Carlo Hospital (protocol number 0008332) and informed consent was obtained according to Italian regulations. The study has been registered on ClinicalTrials.gov.

### Study design

**Fig. 1:**
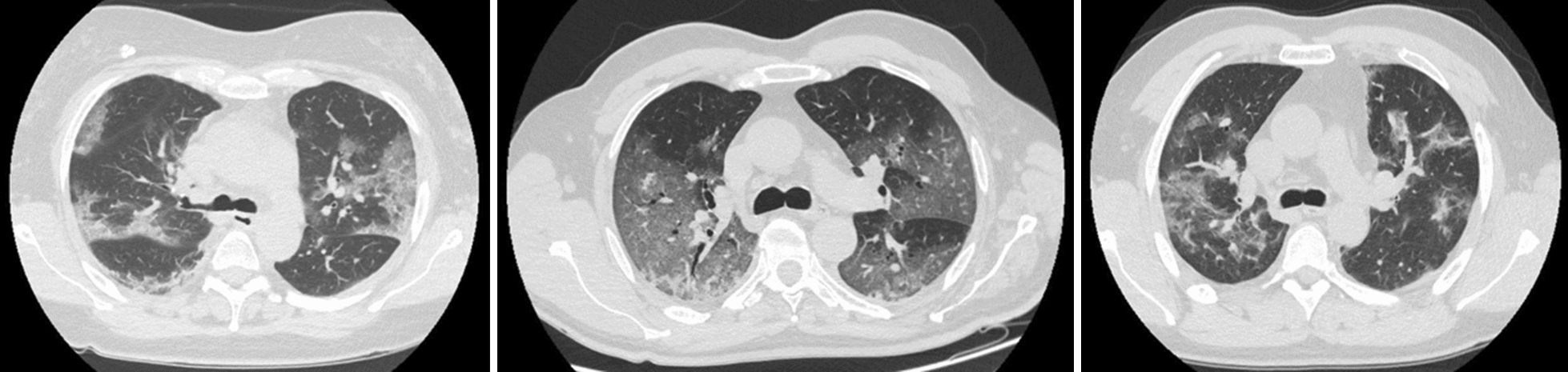
Lung Computed Tomography. Lung Computed Tomography images (at the level of the carina), extracted from lung CT of three different patients, representative of the ARDS severity of the study population

At hospital admission every patient received a CT of the lung at atmospheric pressure (Fig. 1).

CPAP was delivered through a high flow generator (VitalSigns inc., Totowa, USA; 90–140 L/min; Myo 3133A, Pulmodyne), using a helmet (Starmed, Italy). PEEP within the helmet was maintained between 7.5 and 10 cmH_2_O through an adjustable mechanical valve and FiO_2_ was titrated to maintain a SpO_2_ > 94%.

When required to improve collaboration, patients received light sedation/analgesia with continuous infusion of morphine titrated between 10 and 20 mg/24 h and/or hydroxyzine titrated between 50 and 100 mg three times a day (150–300 mg/24 h), and haloperidol, in case of delirium, titrated between 0.5 mg four times a day and 2 mg six times a day (2–12 mg/24 h). The sedation was already started at study enrollment in supine position and the level of sedation (i.e., amount of used drugs) was not changed throughout the study, among supine and prone position.

At enrollment, a standard balloon catheter was positioned to measure the esophageal pressure (Nutrivent, SIDAM Italy) and a single-use padset sensor was placed on chest to measure respiratory volumes (ExSpiron 1Xi system, Respiratory Motion Inc., Inspired Innovation, Watertown, MA). On supine position and after 3 h from the initiation of prone position, arterial blood gas analysis, hemodynamics and esophageal pressure swings were assessed. The tidal volume, respiratory rate and minute ventilation were measured and breathing comfort was evaluated.

### Data collection

Patient’s demographic data and the most relevant clinical characteristics were recorded at study entry. HACOR and Charlson score were also computed [24, 25].

To measure the esophageal pressure a standard balloon catheter, consisting of a 108 cm long tube with a 10 cm long walled balloon, was positioned in the lower section of the esophagus. The esophageal catheter was emptied of air and introduced transorally into the esophagus to reach a depth of between 45 and 55 cm measured from the mouth. Subsequently the balloon was inflated with 4 ml of air [26]. The amount of gas in the balloon was periodically checked throughout the experiment. The esophageal pressure was measured by the OptiVent™ monitor (Sidam S.R.L., Modena, Italy), a device for the automated management of the esophageal catheter.

The tidal volume, respiratory rate and minute ventilation were measured by the ExSpiron 1Xi system. This system employs a bioelectrical impedance technique and consists of a single-use PadSet sensor placed on the chest [27].

The presence of dyspnea was evaluated on the basis of the “Borg perceived dyspnea” scale [28]. Patients were asked “how short of breath are you?” according to a scale from 0 to 10, zero indicating no shortness of breath and ten an extremely severe dyspnea (Additional file 1: Fig. S1). Patients were instructed on the appropriate use of the scale before the beginning of the protocol.

The non-invasive work of breathing was objectively evaluated by an independent physician according to a dedicated scale for COVID-19 patients, ranging from 1 to 7, considering the respiratory rate, the presence of nasal flaring and the use of sternocleidomastoid as well as abdominal muscles [29].

Patients were followed until hospital discharge, the occurrence of intubation and/or death were recorded.

### Data analysis

A quantitative CT scan analysis was performed. The total lung gas volume, weight, and the proportions of the respective inflation compartment (not inflated, poorly inflated, well inflated and over inflated) were computed as previously described [30].

Breath patterns were continuously recorded for 3–5 min, then analyzed and the results averaged for each study period.

As surrogate measure for the inspiratory effort the esophageal pressure swing (ΔPes), that is the maximal negative inspiratory deflection of esophageal pressure during the inspiration, was used. In addition, the dynamic transpulmonary pressure (dTPP) during the inspiration, that is the maximal difference between the airway pressure and esophageal pressure, the modified pressure time product (mPTP), computed as the product of ΔPes and respiratory rate, and the work of breathing (WOB), calculated as the product of tidal volume, respiratory rate and the dynamic transpulmonary pressure, were used as inspiratory effort surrogate measures [20, 22]. For the computation of the dynamic transpulmonary pressure (dTPP), the airway pressure was not measured, as it was assumed to be equal to the applied external PEEP by the mechanical valve of the helmet [31]. Dynamic lung compliance was computed as the quotient between tidal volume and ΔPes. The chest wall elastance had to be neglected due to the impossibility to perform an airway occlusion maneuver in a spontaneously breathing patient.

The ventilatory ratio and the estimated dead space fraction were computed according to previously described equations (Additional file 1: Annex S1).

### Endpoints

The main endpoint was the change in work of breathing from supine to prone position, whereas changes in oxygenation and comfort of breathing represented secondary endpoints.

### Statistical analysis

A sample size of 40 patients was chosen given the mechanistic design of this study, in accordance to similar investigations having studied non-invasively mechanically ventilated patients [20, 22, 32]. Differences in studied lung mechanics and blood gas analytics between supine and prone position were studied employing a linear mixed effects model analysis. As independent variable fixed effect, supine/ prone position was entered into the model. As random effects, intercepts for subjects were employed. P values were calculated using a likelihood ratio test of the full model, with the effect in question, against a “null model”, without the effect in question. P values for fixed effects were obtained by Satterthwaite’s method. Differences between supine and prone position for categorical variables were studied by means of Fisher’s exact test, as pertinent. Bivariate correlation analyses were performed employing Pearson, Spearman’s or Kendall’s correlation, as appropriate. Statistical analysis was performed via a fully scripted data management pathway using the R environment for statistical computing version 4.1.0. A two-sided *p* < 0.05 was considered statistically significant. Values are given as medians with interquartile ranges or counts and percentages as appropriate, while individual variable effects between supine and prone position are reported as mean difference with 95% confidence intervals (CI).

## Results

Forty patients suffering from CARDS and supported with helmet CPAP were enrolled in the study. The main baseline characteristics are shown in Table 1. Patients were mainly of male sex (65%), presented a median age of 59 [53–68] years, a body mass index of 27 [25–31] kg/m^2^ and had a median of 2 [1–3] comorbidities, of which arterial hypertension was the most prominent (40%). They were admitted to the hospital 6 [5–10] days after symptom onset. Helmet CPAP and prone position were started after 2 [2–4] days from hospital admission. All patients received the first pronation at the time of enrollment and were able to maintain the prone position consecutively for 3 h.

**Table 1:** Demographics and baseline characteristics of the study population

	Total population *N* = 40
Age, years	59 [53–68]
Male sex	26 (65)
Body mass index, kg/m^2^	27 [25–31]
Charlson comorbidity index	2 [1–3]
Arterial hypertension	16 (40)
Diabetes mellitus	5 (12)
Solid tumor	4 (10)
Immunosoppression	2 (5)
HACOR score at study start	3 [0–4]
SOFA score at study start	3 [2, 3]
SAPS II score at study start	24 [22–27]
Time from symptoms to hospital admission, days	6 [5–10]
Time from hospital admission to CPAP and proning, days	2 [2–4]
C-reactive protein, mg/L	60 [42–102]
D-dimers, μg/L	371 [248–575]
Lactate, mmol/L	1.3 [1.0–1.4]
ARDS classification	
Mild	14 (35)
Moderate	22 (55)
Severe	4 (10)
PaO_2_/ FiO_2_ ratio, mmHg	166 [136–224]
PaO_2_, mmHg	114 [90–141]
PaCO_2_, mmHg	38 [35–42]
PEEP, cmH_2_O	10 [8–10]
Dynamic lung compliance [ml/cmH_2_O]	64 [42–89]
Requirement for endotracheal intubation	7 (18)
Mortality at 28 days	4 (10)

*HACOR* Heart Rate, Acidosis, Consciousness, Oxygenation, Respiratory Rate, *SOFA* Sequential Organ Failure Assessment, *SAPS II* Simplified Acute Physiology Score, *CPAP* continuous positive airway pressure, *ARDS* acute respiratory distress syndrome, *PEEP* positive end-expiratory pressure

Data are presented as counts (percentages) for dichotomous values or median [interquartile range] for continuous values unless otherwise specified

The applied PEEP with helmet CPAP was 10 [8–10] cmH_2_O with a PaO_2_/FiO_2_ ratio of 166 [136–224] mmHg. At admission the quantitative lung CT scan showed a median lung weight and gas volume of 1112 [903–1291] g and 3282 [2698–3721] ml, respectively. The amount of non-aerated lung tissue corresponded to 11 [8–17] %, while the well-aerated tissue was 52 [43–62] % of the total lung tissue (Fig. 1, Additional file 1: Table S1).

### Gas exchange

**Fig. 2:**
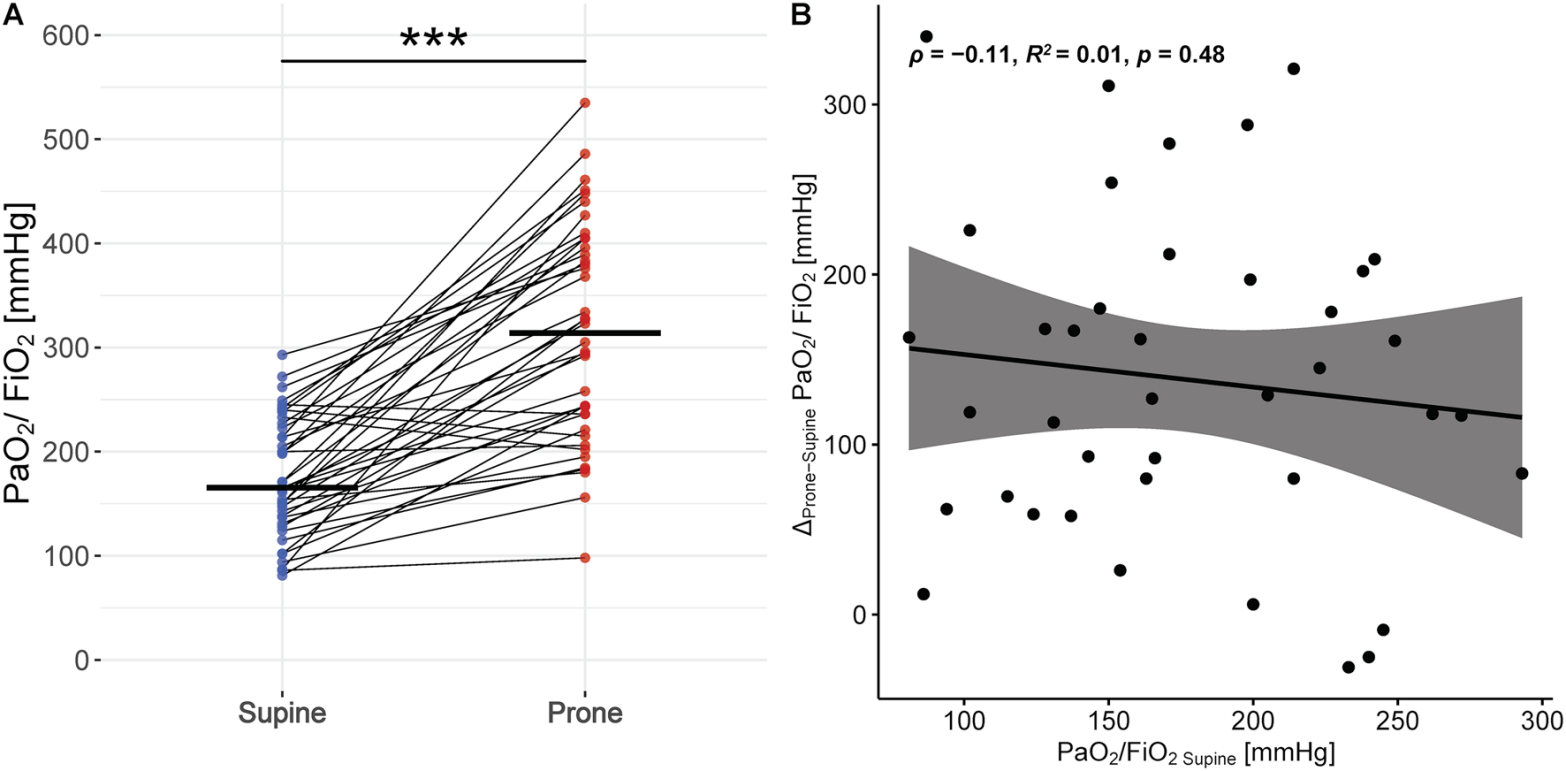
**A** Change in PaO_2_/ FiO_2_ Ratio from supine to prone position. Individual patient measurements are represented by dots (blue representing supine and red prone position, respectively), thin lines connect pairs of individual patient measurements (supine*—*prone), and thick horizontal lines display the median. *Significance levels: P value* ≥ *0.05—NS,* < 0.05—*, < 0.01—**, < 0.001—***. **B** Correlation between the change in PaO_2_/ FiO_2_ Ratio from supine to prone position and its respective value in supine position. The scatter-plot represents individual patient measurement-pairs, the black line displays the fitted linear regression, and the shaded gray area depicts its 95% Confidence Interval. *ρ—Pearson correlation coefficient, p—P value*

Gas exchange data in supine and prone position are shown in Table 2. The PaO_2_/FiO_2_ was higher in prone position compared to supine (Supine: 166 [136–224] mmHg, Prone: 314 [232–398] mmHg, *p* < 0.001). Thirty-four (85%) patients had an improvement in PaO_2_/FiO_2_ higher than 20%, corresponding to a mean rise of 138 [95% CI 108–169] mmHg, from supine to prone position. The change in oxygenation from supine to prone position was not related to the oxygenation in supine (Fig. 2). The baseline CT scan was not associated with the degree of oxygenation improvement (Additional file 1: Figs. S10–S13).

**Table 2:** Respiratory mechanics and gas exchange of the study population during supine position and prone position

	Supine *N* = 40	Prone *N* = 40	*p*	Mean difference^†^ [95% CI]
Respiratory rate, bpm	20 [17 to 24]	17 [15 to 19]	< 0.001	− 3 [− 4 to − 2]
Tidal volume, ml	431 [359 to 498]	424 [379 to 481]	0.478	− 11 [− 43 to − 21]
Tidal volume, ml/kg	6.9 [6.0 to 7.9]	6.9 [5.7 to to 7.9]	0.517	− 0.16 [− 0.65 to 0.33]
Minute ventilation, l/min	8.6 [7.3 to 10.6]	7.7 [6.6 to 8.6]	< 0.001	− 1.4 [− 1.9 to − 0.8]
PaO_2_/ FiO_2_ ratio, mmHg	166 [136 to 224]	314 [232 to 398]	< 0.001	138 [108 to 169]
PaO_2_, mmHg	114 [90 to 141]	206 [143 to 270]	< 0.001	95 [74 to 116]
PaCO_2_, mmHg	38 [3 to 42]	39 [37 to 41]	0.029	1.3 [0.1 to 2.5]
Ventilatory ratio	1.4 [1.3 to 1.6]	1.3 [1.1 to 1.5]	< 0.001	− 0.16 [− 0.25 to − 0.07]
Estimated dead-space fraction, %	41 [3 to 49]	32 [23 to 39]	< 0.001	− 9 [− 14 to − 5]

*CI* confidence interval, *bpm* breaths per minute

Data are presented as median [interquartile range] for continuous values unless otherwise specified

^†^Supine Position taken as reference

Arterial carbon dioxide slightly increased between supine and prone position. The ventilatory ratio and the estimated physiological dead space decreased from supine to prone position (Table 2, Additional file 1: Figs. S2 and S3).

### Respiratory parameters, work and comfort of breathing

The respiratory rate and minute ventilation decreased from supine to prone position from 20 [17–24] to 17 [15–19] 1/min and 8.6 [7.3–10.6] to 7.7 [6.6–8.6] L/min, respectively (Table 2).

**Fig. 3:**
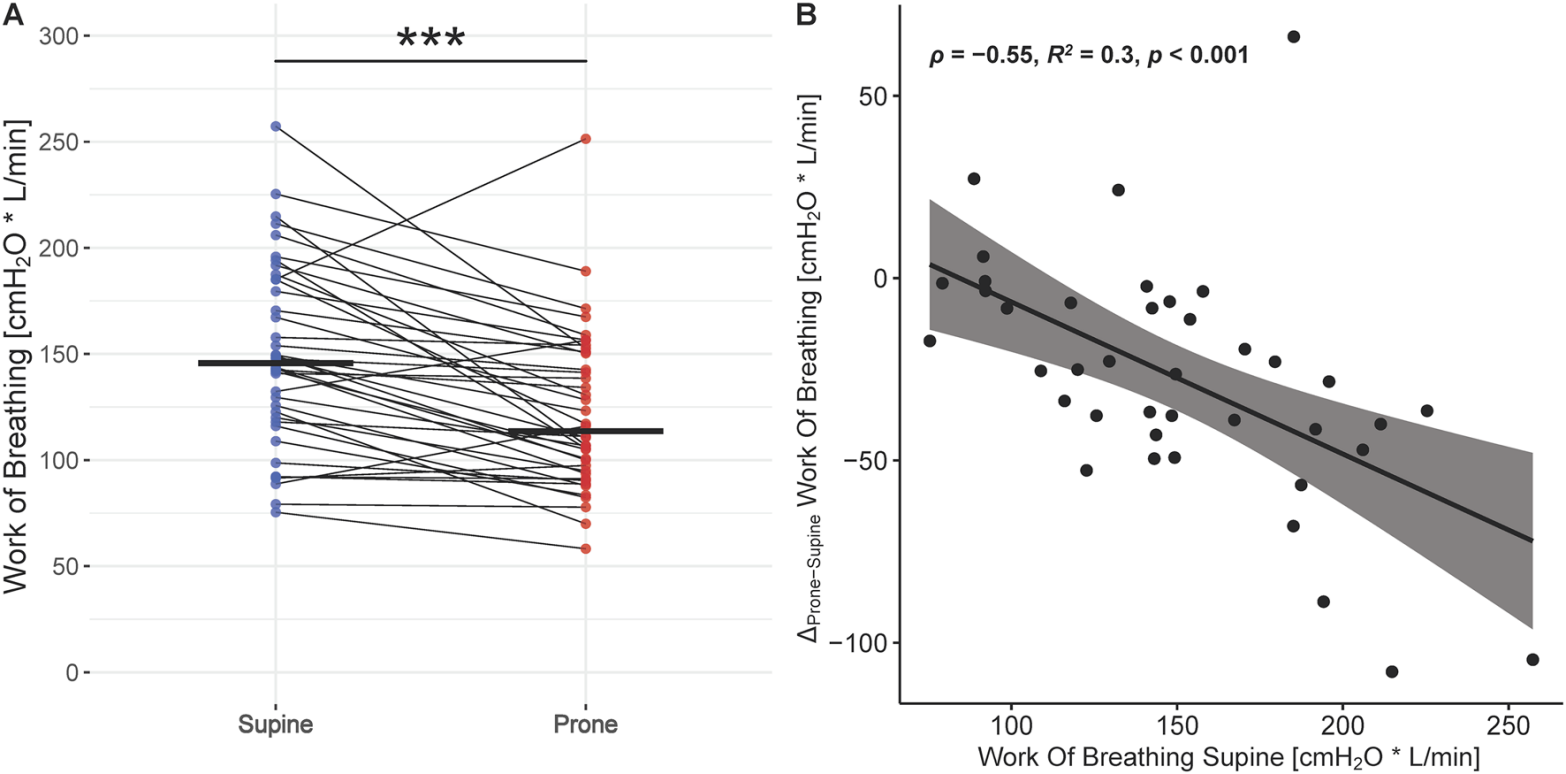
**A** Change in Work of Breathing from supine to prone position. Individual patient measurements are represented by dots (blue representing supine and red prone position, respectively), thin lines connect pairs of individual patient measurements (supine*—*prone), and thick horizontal lines display the median. *Significance levels: P value* ≥ *0.05—NS,* < 0.05—*, < 0.01—**, < 0.001—***. **B** Correlation between the change in Work of Breathing from supine to prone position and its respective value in supine position. The scatter-plot represents individual patient measurement-pairs, the black line displays the fitted linear regression, and the shaded gray area depicts its 95% Confidence Interval. *ρ—Pearson correlation coefficient, p—P value*

**Fig. 4:**
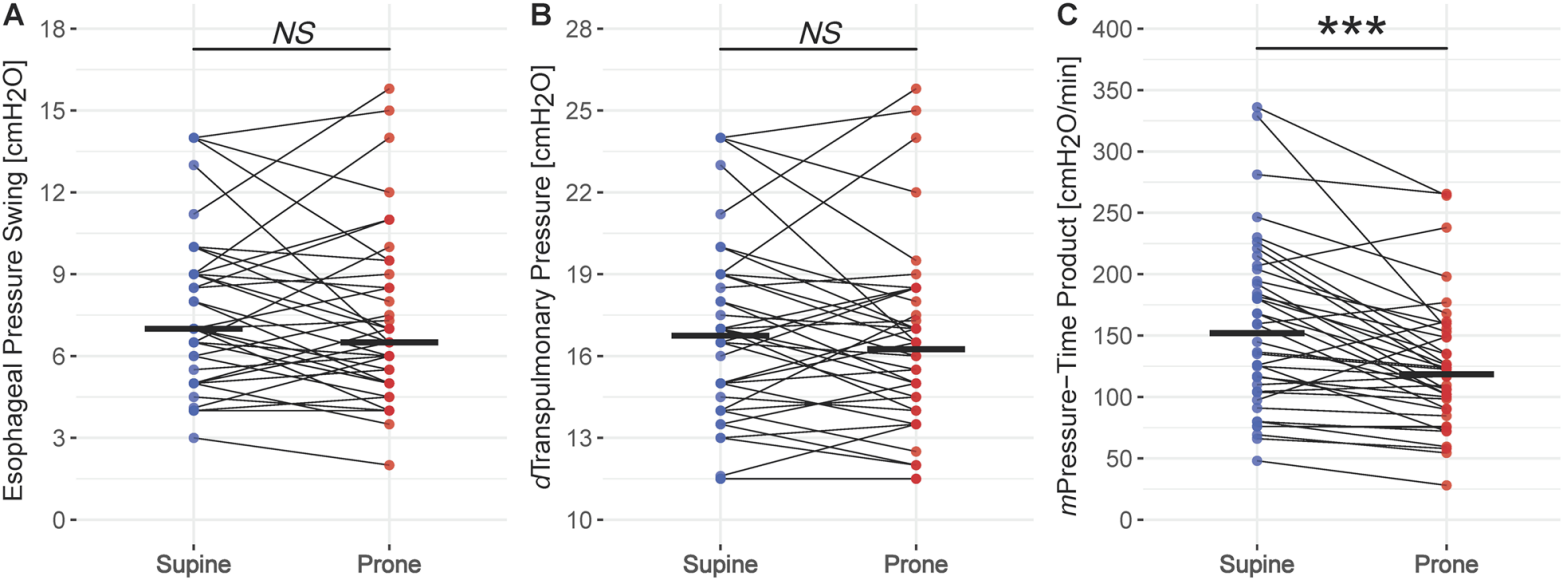
Changes in Esophageal Pressure Swing (**A**), dynamic Transpulmonary Pressure (**B**) and modified Pressure–Time Product (**C**) between supine and prone position. Individual patient measurements are represented by dots (blue representing supine and red prone position, respectively), thin lines connect pairs of individual patient measurements (supine*—*prone), and thick horizontal lines display the median. *Significance levels: P value* ≥ *0.05—NS,* < 0.05—*, < 0.01—**, < 0.001—***

The prone position did not have any effect on ΔPes (*p* = 0.306) and dTPP (*p* = 0.34) (Table 3). Conversely, the mPTP and the WOB decreased from 152 [104–197] to 118 [90–150] cmH_2_O/min and from 146 [120–185] vs 114 [95–151] cmH_2_O*L/min, respectively (Table 3, Figs. 3 and 4). Thirty-three (83%) patients experienced a reduction in the WOB from supine to prone position, while 26 (65%) experienced a reduction of greater than or equal to 10%. The reduction in the WOB was highly correlated to the baseline in supine position, which was the only marker of inspiratory effort, among esophageal pressure swing, dynamic transpulmonary pressure and modified pressure time product, with prognostic capacity to predict a reduction in the same (*p* < 0.001) (Fig. 3, Additional file 1: Fig. S4). The respiratory rate in supine position was not correlated to the reduction in the WOB from supine to prone position (Additional file 1: Fig. S5).

**Table 3:** Extended respiratory mechanics and clinical assessment of the study population during supine position and prone position

	Supine *N* = 40	Prone *N* = 40	*p*	Mean difference^†^ [95% CI]
Esophageal pressure swing, cmH_2_O	− 7 [− 9 to − 5]	− 6 [− 9 to − 5]	0.306	0.4 [− 0.4 to 1.1]
Dynamic transpulmonary pressure, cmH_2_O	17 [14 to 19]	16 [14 to 18]	0.34	− 0.4 [− 1.1 to 0.4]
Modified pressure–time product, cmH_2_O/min	152 [104 to 197]	118 [90 to 150]	< 0.001	− 33 [− 47 to − 19]
Work of breathing, cmH_2_O * L /min	146 [120 to 185]	114 [95 to 151]	< 0.001	− 27 [− 38 to − 17]
Borg Dyspnea Scale 0—none 1—very light 2—light 3—moderate 4—rather intense	29 (72) 3 (8) 5 (12) 1 (2) 2 (5)	37 (92) 2 (5) 1 (2) 0 (0) 0 (0)	0.005	− 0.5 [− 0.8 to − 0.2]
Non-invasive Work of Breathing Scale 1 2 3 4	20 (50) 12 (30) 5 (12) 3 (8)	31 (78) 8 (20) 1 (2) 0 (0)	0.001	− 0.5 [− 0.9 to − 0.2]

*CI* confidence interval

Data are presented as median [interquartile range] for continuous values unless otherwise specified

^†^Supine Position taken as reference

The mPTP and the WOB were not related to the CT lung anatomical characteristics (lung gas volume and lung weight) (Additional file 1: Figs. S6–S9). In addition, the CT lung anatomical characteristics were not predictive for the physiological response to proning (Additional file 1: Figs. S10–S13).

Twenty-seven patients reported a sensation of dyspnea in supine position, assessed by the Borg scale, ranging from light to rather intense, which notably decreased and disappeared in all patients but three patients after initiating prone position (*p* = 0.005) (Table 3, Additional file 1: Fig. S14). The patient dyspnea sensation was neither related to the mPTP nor to the WOB (Additional file 1: Fig. S15).

Changes in Hemodynamics between supine and prone position are shown in Additional file 1: Table S2. Subgroup analyses stratifying patients by PaO_2_/ FiO_2_ ratio, ΔPes, dynamic lung compliance (using median value 64 ml/cmH_2_O) and sensation of dyspnea, in supine position, did not reveal any subgroups with a higher benefit from proning (Additional file 1: Tables S3–S6).

### Outcome

Among the 40 enrolled patients, 7 (18%) required endotracheal intubation and invasive mechanical ventilation, and 4 of these (53%) died within the 28 days ensuing study inclusion.

## Discussion

This study, evaluating the effect of awake prone position in CARDS patients treated with helmet CPAP showed that prone position was associated with: (1) a reduction in the work of breathing, (2) an improvement in oxygenation in 85% of the patients, and (3) a reduced sensation of dyspnea in all patients.

COVID-19 pneumonia is characterized by the presence of hypoxemia, bilateral lung infiltrates and microvascular thrombosis within the lung vasculature, contributing to a profound ventilation–perfusion mismatch [33, 34]. Thus, COVID-19, in its most severe expression, is characterized by the same pathophysiologic characteristics that are pathognomonic of ARDS. Awake prone position in non-intubated spontaneously breathing patients with acute respiratory failure, has been suggested to improve oxygenation and was feasible in up to 95% of patients [35, 36]. Several small observational studies in patients suffering COVID-19, have reported similar results in awake spontaneous breathing patients, leading to a recent consensus statement for the management of COVID-19 pneumonia having agreed on the role of prone position as adjunct therapy for the treatment of patients requiring supplement oxygenation to maintain oxygen saturation above 90% [12, 14, 15].

In addition to prone position, non-invasive respiratory support, such as HFNC, helmet CPAP and pressure support have been applied both to ameliorate gas exchange and to reduce the inspiratory effort. The combination of prone position and non-invasive respiratory support has been reported to ameliorate the oxygenation in the majority of the studies [12, 14, 16]. The largest series published by Coppo et al. [12], which enrolled 56 patients most of them with helmet CPAP, showed that oxygenation improved on average by more than 50%. In the present study the oxygenation similarly improved, independently of their baseline degree of hypoxemia, just after 3 h of pronation. This suggests that pronation can be used as an effective strategy capable to ameliorate gas exchange as a rescue or long-term treatment.

Several beneficial mechanisms associated with prone position previously described both in acute respiratory failure and in ARDS, could be also found in patients suffering CARDS [37]. In line with previous descriptions, in the early phase of the disease, the lung weight and lung gas volume of patients with CARDS was approximately half and double, respectively, to that observed in classic ARDS [38]. In addition, the amount of non-aerated and poorly aerated lung tissue was quite low. This may indicate that the impairment of oxygenation in the early stage of CARDS is mainly governed by an impairment of the lung perfusion due to a pronounced endothelitis and microvascular thrombosis [39, 40]. Although we did not measure lung perfusion, the improvement in oxygenation and dead space associated with prone position could be mainly an effect of pulmonary ventilation and perfusion redistribution [34, 37, 41].

In addition to hypoxemia, CARDS is characterized by vigorous breathing efforts and a high respiratory rate [34]. The strong inspiratory efforts generate high levels of work of breathing and thus, excessive stress and strain to the lung parenchyma, increasing pulmonary edema and exacerbating the lungs injury. These factors in addition to the natural course of lung disease, if not corrected, might promote muscular exhaustion and a deterioration of the lungs function, frequent causes of treatment failure [42].

To date, no data exists on the effects of awake prone position with helmet CPAP on the work of breathing in CARDS. In the present study the inspiratory effort exerted by the respiratory muscles assessed by means of the changes in esophageal pressure and dynamic transpulmonary pressure did not vary between supine and prone position, while the modified pressure time product and work of breathing significantly decreased. These beneficial effects were related mainly to a reduction in respiratory rate, while the tidal volume did not change, thus arguing against a significant change in pulmonary viscoelastic properties, or alveolar recruitment, and diaphragm position and function, between supine and prone position. Furthermore, the reduction in work of breathing was correlated to the amount of work of breathing in supine position, suggesting a higher utility of prone position in patients presenting elevated inspiratory efforts [43].

It has been reported that between 15 and 31% of spontaneously breathing and non-invasively respiratory supported patients suffering COVID-19 subjectively perceive dyspnea [12, 44]. In the present study 32% of the patients described the presence of dyspnea, ranging from light to moderately severe according to the Borg dyspnea scale. Most importantly, dyspnea was not related to the severity of hypoxemia, level of carbon dioxide or work of breathing, this could suggest a blunting of the respiratory centers by SARS-COV-2 as has been previously suggested [33, 45]. A similar dissociation between the comfort of breathing and work of breathing has been previously described in patients suffering ARDS during the weaning phase from invasive mechanical ventilation [28]. Interestingly, prone position was associated with an improved comfort of breathing.

Prone position delivered concomitantly to helmet CPAP thus presents unique advantages regarding improvements in gas exchange and a reduction in the work of breathing, while its impact on endotracheal intubation and invasive mechanical ventilation requirements nevertheless remain unclear [15, 23].

### Limitations

Possible limitations of this study are: (1) long term effects of prone position and persistency of effects after returning to supine position are unclear, (2) trans-diaphragmatic pressure was not measured, thus the possible effect of expiratory muscle activity on the measured pressure cannot be assessed, (3) due to the study design, effects of prone position on clinically relevant outcomes could not be evaluated, (4) prone position was not randomized.

In conclusion, awake prone position in addition to helmet CPAP may be offered to CARDS patients to improve oxygenation and reduce WOB. However, a strict clinical evaluation should prevail, to prevent delays in endotracheal intubation and provision of invasive mechanical ventilation.

## Supplementary Information

**Additional file 1.** Tables S1–S6, Annex S1 and Figures S1–S15.

## Data Availability

The data sets used and/or analysed during the current study are available from the corresponding author on reasonable request.

## Abbreviations

ARDS: Acute respiratory distress syndrome
CARDS: COVID-19-associated ARDS
CI: Confidence interval
COVID-19: Coronavirus 2019 disease
CPAP: Continuous positive airway pressure
CT: Computer tomography
ΔPes: Esophageal pressure swing
dTPP: Dynamic transpulmonary pressure
HACOR: Heart Rate, Acidosis, Consciousness, Oxygenation, Respiratory Rate
mPTP: Modified pressure time product
PEEP: Positive end-expiratory pressure
PSILI: Patient self-inflicted lung injury
SAPS II: Simplified Acute Physiology Score
SOFA: Sequential Organ Failure Assessment
VILI: Ventilation induced lung injury
WOB: Work of breathing
