# Supplementary material for: Awake prone position reduces work of breathing in patients with COVID-19 ARDS supported by CPAP
Source: Ann Intensive Care. 2021 Dec 20;11:179. doi: 10.1186/s13613-021-00967-6 (PMC8686083; doi:10.1186/s13613-021-00967-6)
Supplement: Supplementary file 1 — Additional file 1. Tables S1–S6, Annex S1 and Figures S1–S15. [file 13613_2021_967_MOESM1_ESM.docx]

**Additional file**

**Effects of Awake Prone Position in Helmet CPAP Supported COVID-19 ARDS Patients**

Davide Chiumello, Elena Chiodaroli, Silvia Coppola, Simone Cappio Borlino, Claudia Granata, Matteo Pitimada, Pedro D Wendel Garcia.

- **e-Figure 1.** Borg Dyspnea Scale.
- **e-Annex 1.** Measurements and Formulas.
- **e-Table 1.** CT variables of the Study Population.
- **e-Table 2.**Hemodynamics of the Study Population during supine position and after 3 Hours of prone position.
- **e-Figure 2.** Changes in PaCO_2_ and estimated Dead Space Fraction between supine and prone position.
- **e-Figure 3.** Changes in Respiratory Rate and Tidal Volume between supine and prone position.
- **e-Figure 4.** Correlation plots between the change in Esophageal Pressure Swings, dynamic Transpulmonary Pressure and modified Pressure-Time Product from supine to prone position and their respective value in supine position.
- **e-Figure 5.** Correlation plots between the PaO_2_/ FiO_2_ Ratio, Esophageal Pressure Swing, modified Pressure-Time Product and Work of Breathing, respectively and the Total Lung Gas Volume in supine position.
- **e-Figure 6.** Correlation plots between the PaO_2_/ FiO_2_ Ratio, Esophageal Pressure Swing, modified Pressure-Time Product and Work of Breathing, respectively and the Total Lung Weight in supine position.
- **e-Figure 7.** Correlation plots between the PaO_2_/ FiO_2_ Ratio, Esophageal Pressure Swing, modified Pressure-Time Product and Work of Breathing, respectively and the Percentage of Non-aerated lung tissue in supine position.
- **e-Figure 8.** Correlation plots between the PaO_2_/ FiO_2_ Ratio, Esophageal Pressure Swing, modified Pressure-Time Product and Work of Breathing, respectively and the Percentage of Well-aerated lung tissue in supine position.
- **e-Figure 9.** Correlation plots between the change in PaO_2_/ FiO_2_ Ratio, Esophageal Pressure Swings, modified Pressure-Time Product and Work of Breathing from supine to prone position and the Percentage of Non-aerated lung tissue in supine position.
- **e-Figure 10.** Correlation plots between the change in PaO_2_/ FiO_2_ Ratio, Esophageal Pressure Swings, modified Pressure-Time Product and Work of Breathing from supine to prone position and the Percentage of Well-aerated lung tissue in supine position.
- **e-Figure 11.** Change in distribution of the non-invasive Work of Breathing Scale and the Borg Dyspnea Scale from supine to prone position.
- **e-Figure 12.** Correlation plots between the Borg Dyspnea Scale and the modified Pressure-Time Product and Work of Breathing, respectively in supine position.
- **e-Table 3.** Subgroup Analysis for PaO_2_/ FiO_2_ >150 mmHg and ≤150 mmHg**.**
- **e-Table 4.** Subgroup Analysis for Esophageal Pressure Swing >7 cmH_2_O and ≤7 cmH_2_O.
- **e-Table 5.** Subgroup Analysis for Dynamic Lung Compliance >40 ml/cmH_2_O and ≤40 ml/cmH_2_O.
- **e-Table 6.** Subgroup Analysis for Subjective Sensation Dyspnea versus None.


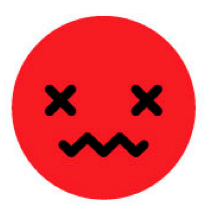

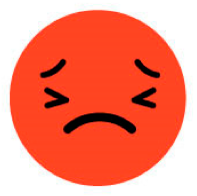

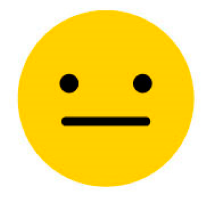

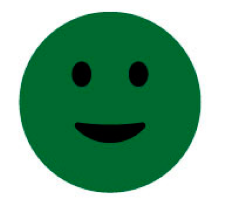

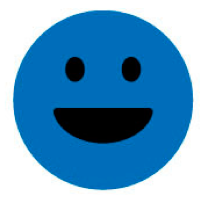
**e-Figure 1. Borg Dyspnea Scale.** Scale to assess “how short of breath” the patient subjectively feels, according to a scale from 0 (no dyspnea at all) to 10 (extremely severe dyspnea)

| **0** | **None** |
| --- | --- |
| **0.5** | **Very, Very Light Dyspnea** |
| **1** | **Very Light Dyspnea** |
| **2** | **Light Dyspnea** |
| **3** | **Moderate Dyspnea** |
| **4** | **Rather Severe Dyspnea** |
| **5** | **Severe Dyspnea** |
| **6** |  |
| **7** | **Very Severe Dyspnea** |
| **8** |  |
| **9** | **Very, Very Severe Dyspnea** |
| **10** | **Extremely Severe Dyspnea** |

**e-Annex 1. Measurements and Formulas.** Esophageal Pressure Swing (ΔPes) measurement.

Formulas for the dynamic Transpulmonary Pressure (dTPP), modified Pressure-Time Product (mPTP), Work of Breathing (WOB), Ventilatory Ratio (E1) and estimated Dead Space Fraction (E2).


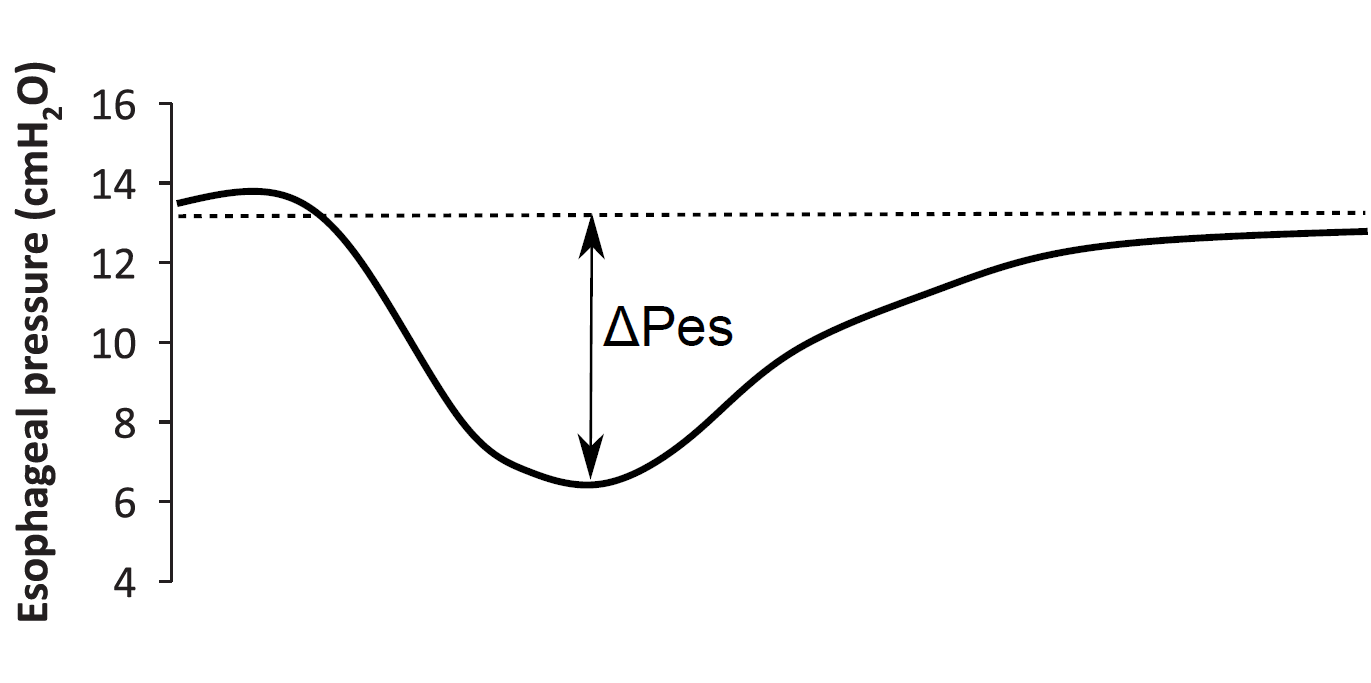


ΔPes = Maximal negative inspiratory deflection of esophageal pressure during the inspiration

$$\boldsymbol{dTPP= Positive End-Expiratory Pressure}\left[ \boldsymbol{cm}\boldsymbol{H}_{\boldsymbol{2}}\boldsymbol{O} \right]\boldsymbol{- \Delta Pes [cm}\boldsymbol{H}_{\boldsymbol{2}}\boldsymbol{O]}$$

$$\boldsymbol{mPTP= \Delta Pes}\left[ \boldsymbol{cm}\boldsymbol{H}_{\boldsymbol{2}}\boldsymbol{O} \right]\boldsymbol{\times Respiratory Rate [bpm]}$$

$$\boldsymbol{WOB=dTPP}\left[ \boldsymbol{cm}\boldsymbol{H}_{\boldsymbol{2}}\boldsymbol{O} \right]\boldsymbol{\times Respiratory Rate [bpm]\times Tidal Volume}\boldsymbol{[L]}$$

$$\boldsymbol{Ventilatory Ratio=}\frac{\boldsymbol{Minute Ventilation}_{\boldsymbol{measured}}\left[ \frac{\boldsymbol{mL}}{\boldsymbol{min}} \right]\boldsymbol{\times}\boldsymbol{PaCO}_{\boldsymbol{2 measured}}\boldsymbol{[mmHg]}}{\boldsymbol{Minute Ventilation}_{\boldsymbol{predicted}}\left[ \frac{\boldsymbol{mL}}{\boldsymbol{min}} \right]\boldsymbol{\times}\boldsymbol{PaCO}_{\boldsymbol{2 ideal}}\boldsymbol{[mmHg]}}$$

$${Minute Ventilation}_{predicted}=Predicted Body Weight \times100 \left[ \frac{mL}{min} \right]$$

${PaCO}_{2 ideal}=37.5 mmHg$

$$\boldsymbol{Estimated Physiological Dead Space=1-}\frac{\boldsymbol{0.863\times}{\dot{\boldsymbol{V}}\boldsymbol{CO}}_{\boldsymbol{2}}}{\boldsymbol{Respiratory Rate [bpm]\times Tidal Volume [L]\times}\boldsymbol{PaCO}_{\boldsymbol{2 measured}}\boldsymbol{[mmHg]}}$$

${\dot{V}CO}_{2}=\frac{Resting Energy Expenditure}{\frac{5.616}{0.8}}+1.584$

${Resting Enery Expenditure}_{Unadjusted Harris-Benedict Estimate-Male}=66.473 + 13.752 \times Weight \left[ kg \right]+5.003\times Height \left[ cm \right]- 6.755\times Age \left[ years \right]$

${Resting Enery Expenditure}_{Unadjusted Harris-Benedict Estimate-Female}=66.473 + 13.752 \times Weight \left[ kg \right]+5.003\times Height \left[ cm \right]- 6.755\times Age \left[ years \right]$

**e-Table 1. Computed Tomography variables of the Study Population.** Data are presented as median [interquartile range].

|  | Total Population  N = 40 |
| --- | --- |
|  |  |
| Total Lung Weight, g | 1112 [903 - 1291] |
| Total Lung Gas, mL | 2115 [1636 - 2650] |
| Total Lung Volume, mL | 3282 [2698 - 3721] |
| Over aerated lung tissue, % | 1.1 [0.5 – 2.4] |
| Well aerated lung tissue, % | 52 [43 - 62] |
| Poorly aerated lung tissue, % | 33 [27 - 40] |
| Non-aerated lung tissue, % | 11 [8 - 17] |

**e-Table 2. Hemodynamics of the Study Population during supine position and after 3 Hours of prone position.** Definition of abbreviations: CI = confidence interval. Data are presented as median [interquartile range].

|  | Supine  N = 40 | Prone  N = 40 | p | Mean Difference [95% CI] |
| --- | --- | --- | --- | --- |
| Heart Rate, beats per minute | 74 [68 - 85] | 74 [63 - 86] | 0.185 | 2 [-1 - 6] |
| Systolic Arterial Pressure, mmHg | 130 [125 - 140] | 130 [126 - 140] | 0.266 | -2 [-6 - 2] |
| Diastolic Arterial Pressure, mmHg | 80 [75 - 85] | 80 [74 - 84] | 0.385 | 1 [-1 - 4] |
| Mean Arterial Pressure, mmHg | 97 [92 - 102] | 97 [93 - 102] | 1 | 0 [-2 - 2] |

**e-Figure 2. Changes in PaCO_2_ and estimated Dead Space Fraction between supine and prone position.** Individual patient measurements are represented by dots (blue representing supine and red prone position, respectively), thin lines connect pairs of individual patient measurements (supine *‒* prone), and thick horizontal lines display the median. *Significance levels: P-value ≥ 0.05 ‒ NS, <0.05 ‒ *, <0.01 ‒ **, <0.001 ‒ ***.*


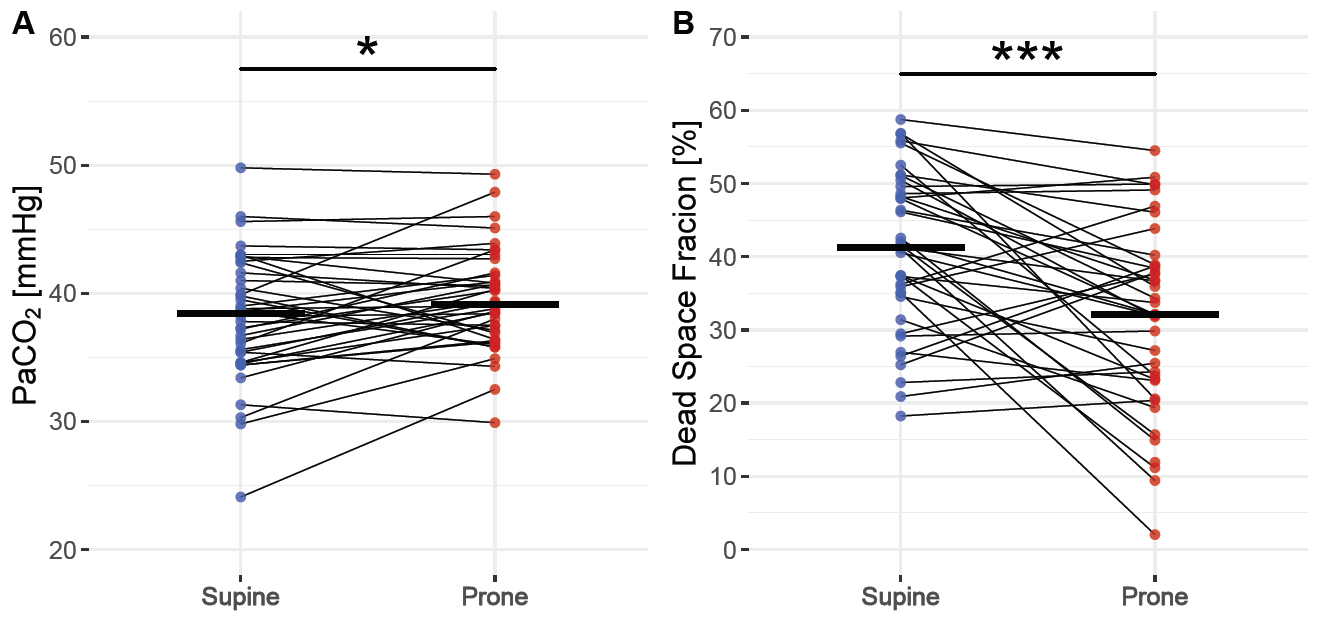


**e-Figure 3. Changes in Respiratory Rate and Tidal Volume between supine and prone position.** Individual patient measurements are represented by dots (blue representing supine and red prone position, respectively), thin lines connect pairs of individual patient measurements (supine *‒* prone), and thick horizontal lines display the median. *Significance levels: P-value ≥ 0.05 ‒ NS, <0.05 ‒ *, <0.01 ‒ **, <0.001 ‒ ***.*


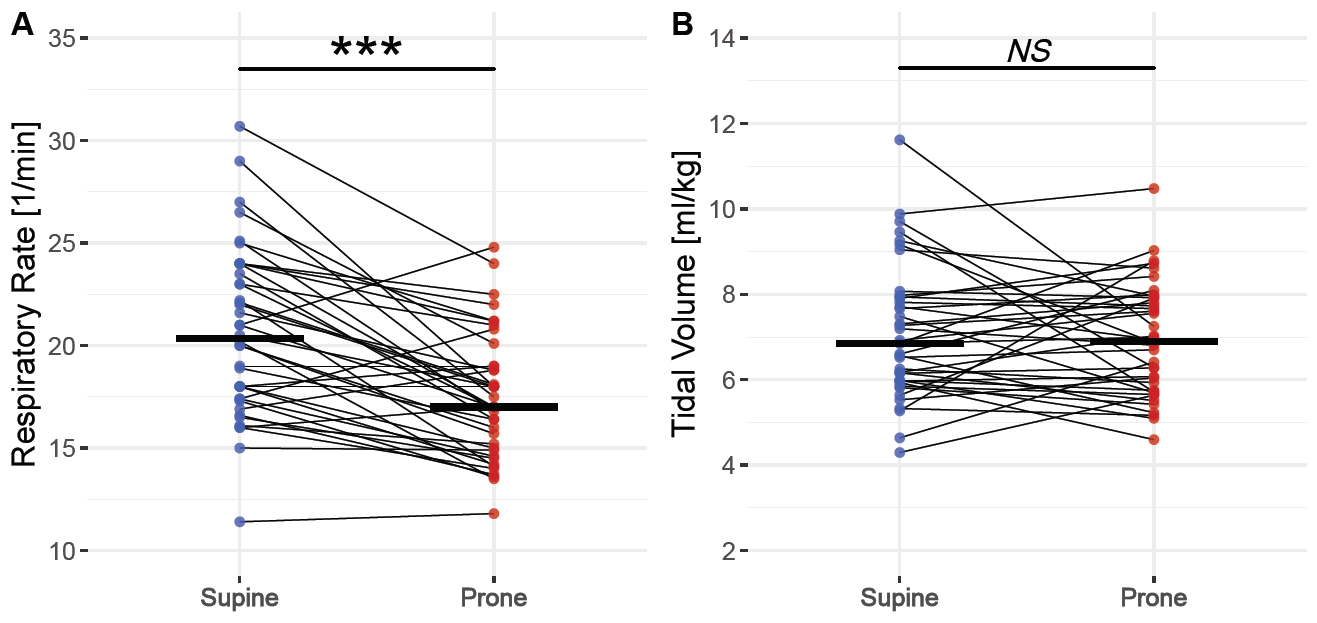


**e-Figure 4. Correlation plots between the change in Esophageal Pressure Swings, dynamic Transpulmonary Pressure and modified Pressure-Time Product from supine to prone position and their respective value in supine position.** The scatter-plot represents individual patient measurement-pairs, the black line displays the fitted linear regression, and the shaded gray area depicts its 95% Confidence Interval. *ρ ‒ Pearson correlation coefficient, p ‒ P-value.*


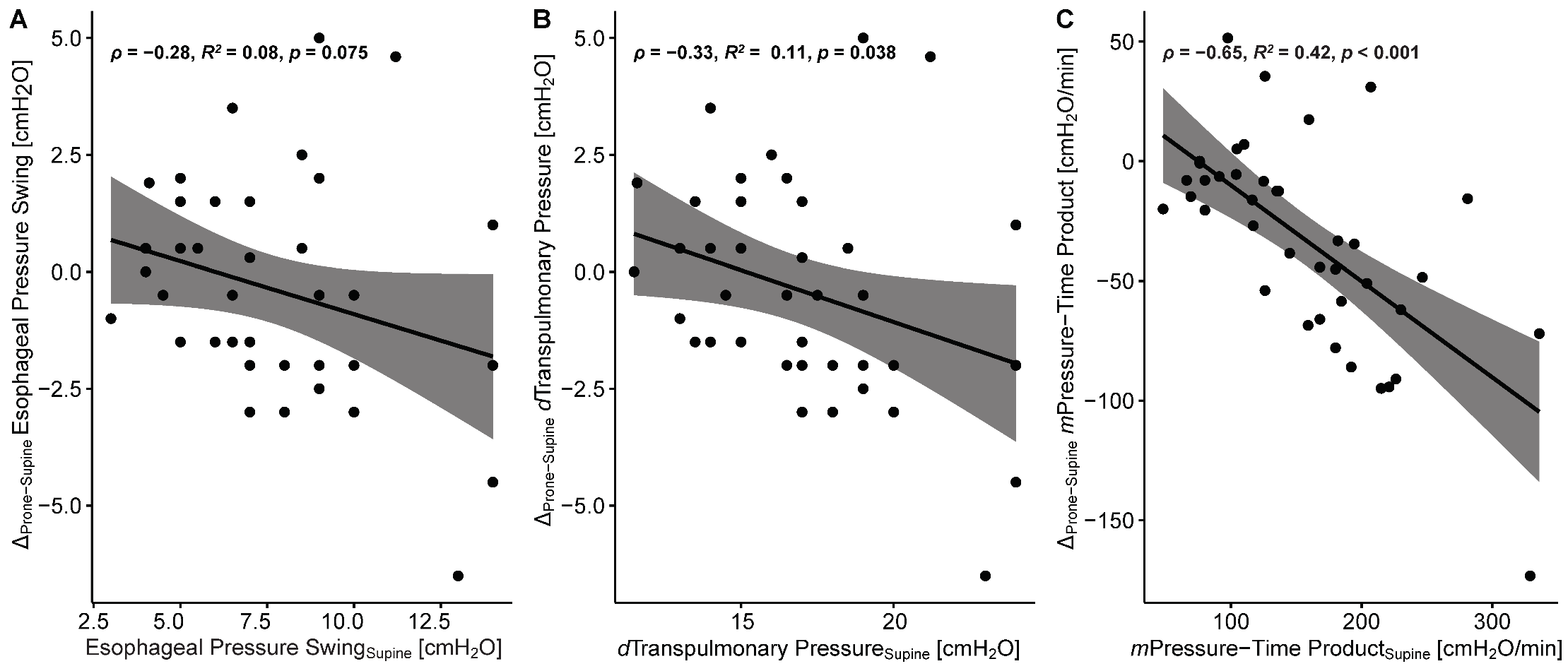


**e-Figure 5. Correlation plots between the change in Work of Breathing from supine to prone position and the respiratory rate in supine position.** The scatter-plot represents individual patient measurement-pairs, the black line displays the fitted linear regression, and the shaded gray area depicts its 95% Confidence Interval. *ρ ‒ Pearson correlation coefficient, p ‒ P-value.*

**
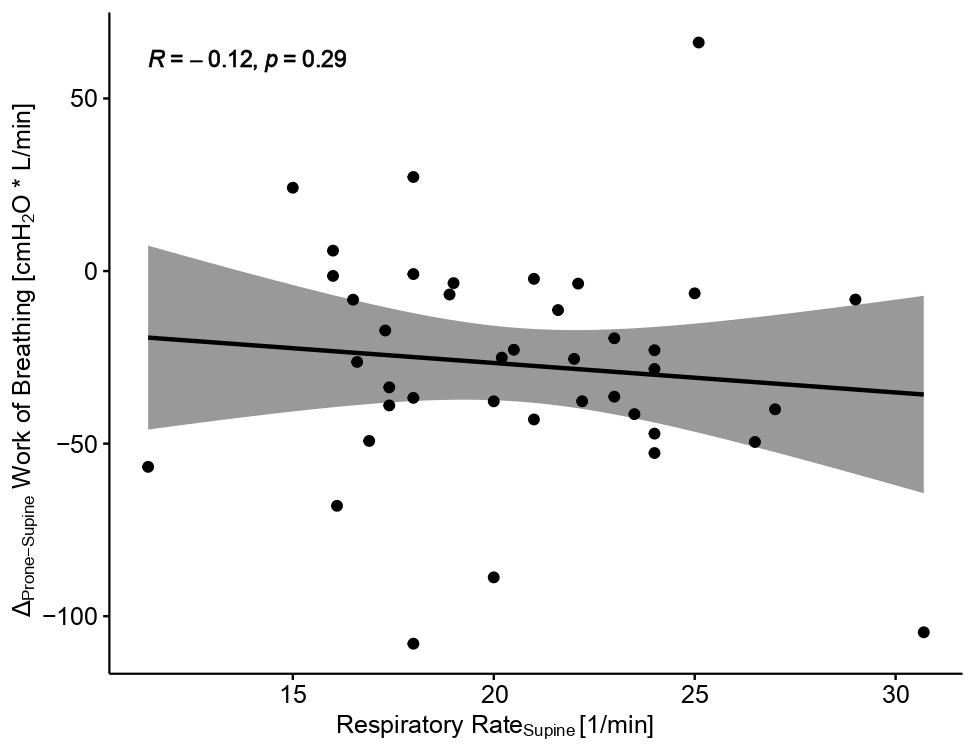
**

**e-Figure 6. Correlation plots between the PaO_2_/ FiO_2_ Ratio, Esophageal Pressure Swing, modified Pressure-Time Product and Work of Breathing, respectively and the Total Lung Gas Volume in supine position.** The scatter-plot represents individual patient measurement-pairs, the black line displays the fitted linear regression, and the shaded gray area depicts its 95% Confidence Interval. *ρ ‒ Pearson correlation coefficient, p ‒ P-value.*


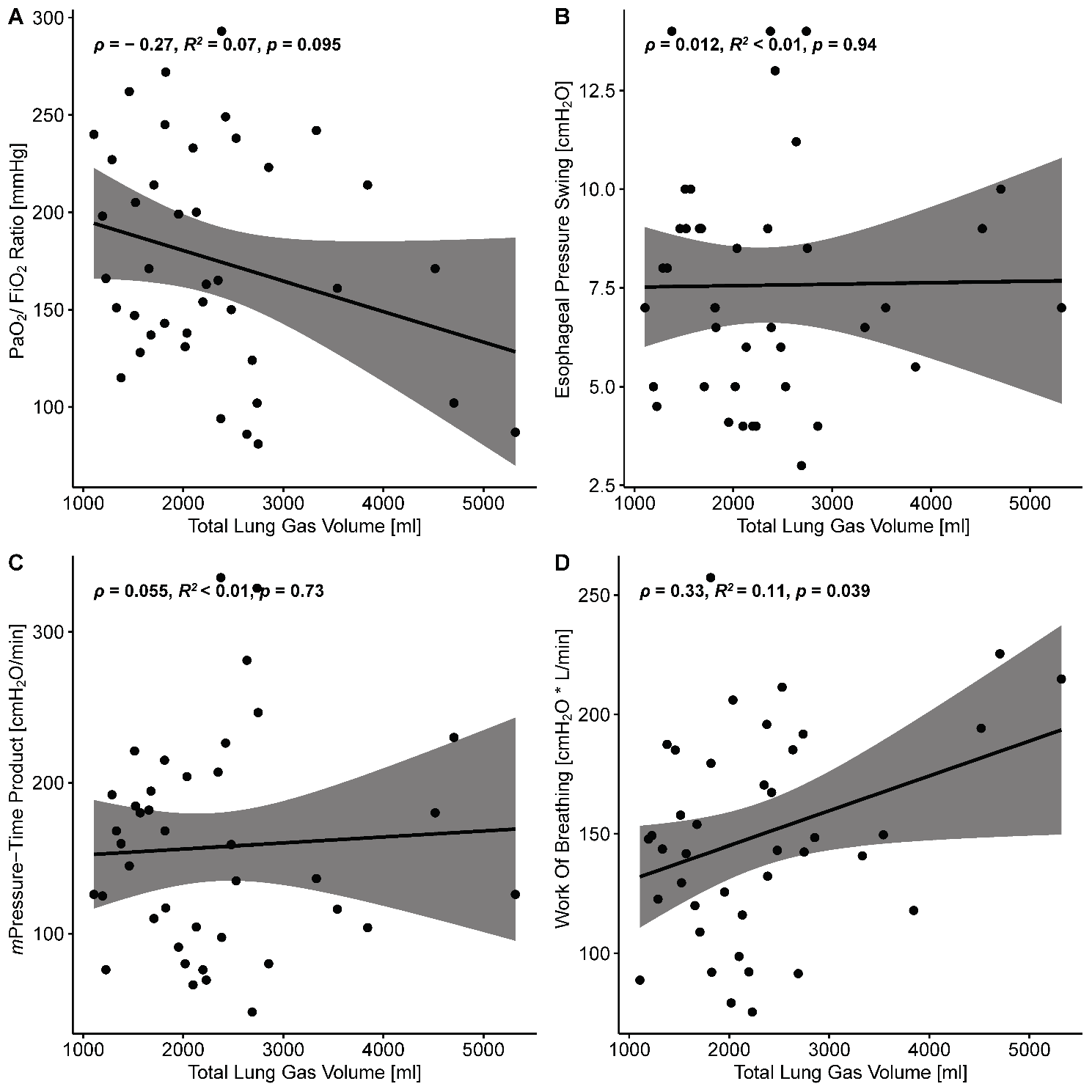


**e-Figure 7. Correlation plots between the PaO_2_/ FiO_2_ Ratio, Esophageal Pressure Swing, modified Pressure-Time Product and Work of Breathing, respectively and the Total Lung Weight in supine position.** The scatter-plot represents individual patient measurement-pairs, the black line displays the fitted linear regression, and the shaded gray area depicts its 95% Confidence Interval. *ρ ‒ Pearson correlation coefficient, p ‒ P-value.*


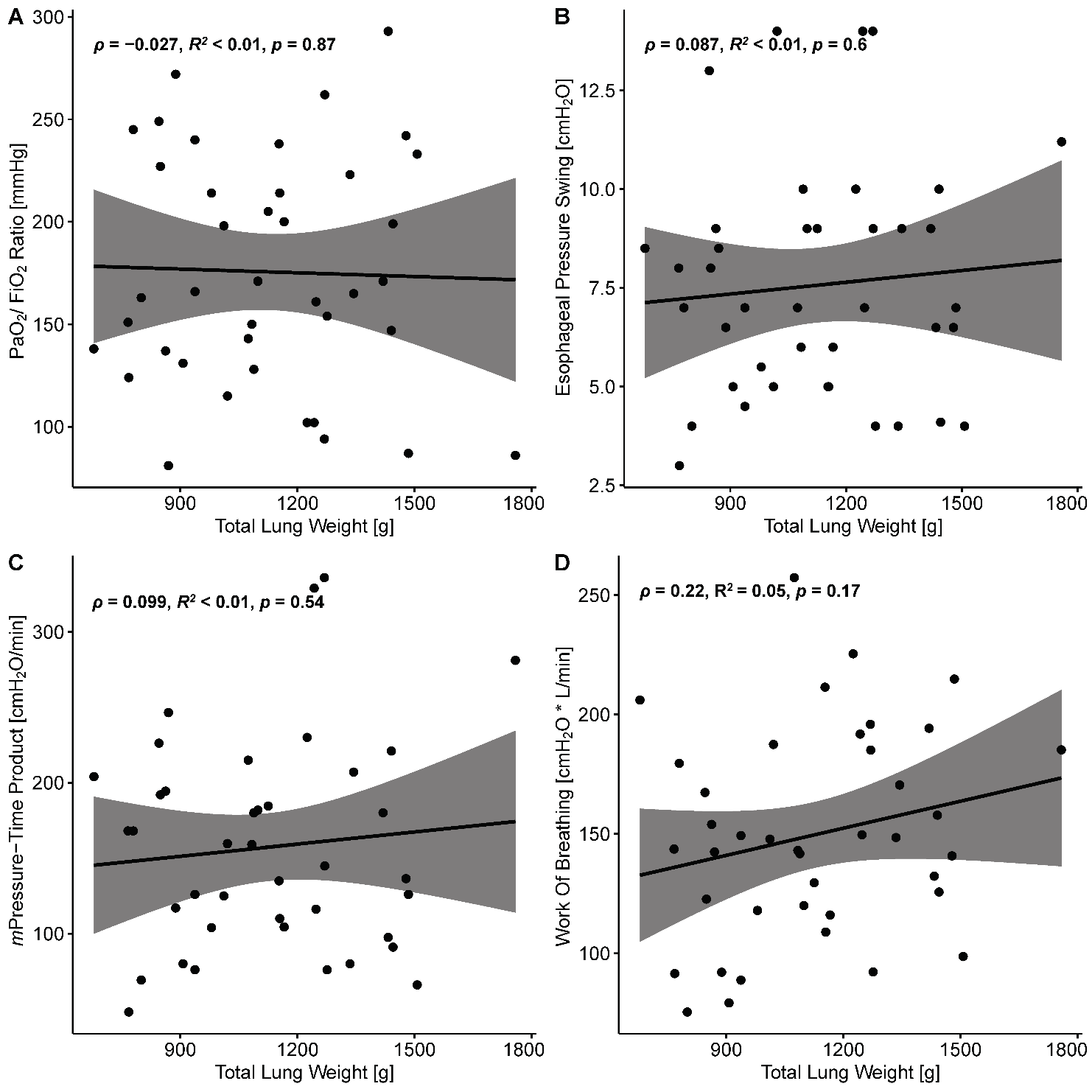


**e-Figure 8. Correlation plots between the PaO_2_/ FiO_2_ Ratio, Esophageal Pressure Swings, modified Pressure-Time Product and Work of Breathing, respectively and the Percentage of Non-aerated lung tissue in supine position.** The scatter-plot represents individual patient measurement-pairs, the black line displays the fitted linear regression, and the shaded gray area depicts its 95% Confidence Interval. *R ‒ Pearson correlation coefficient, p ‒ P-value.*


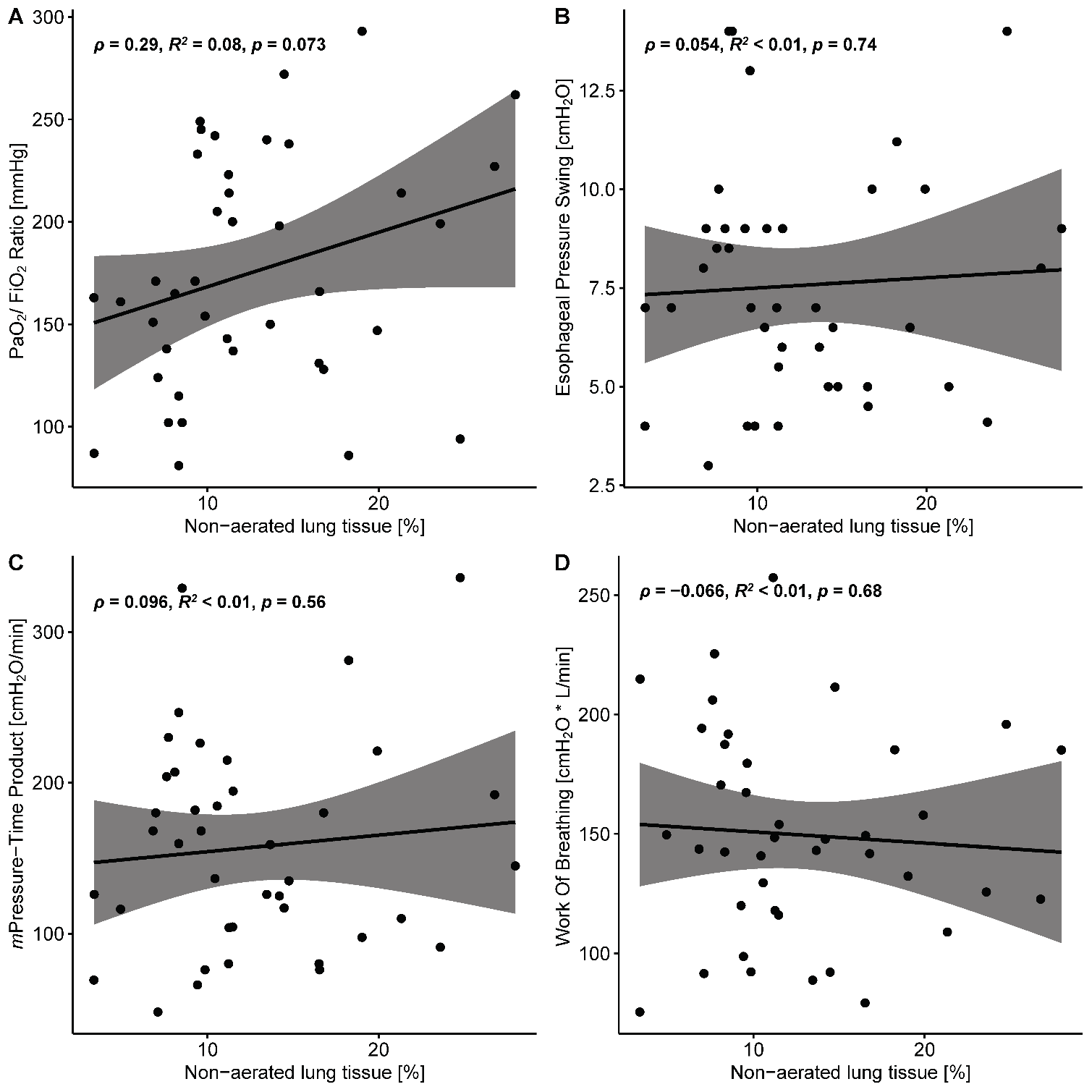


**e-Figure 9. Correlation plots between the PaO_2_/ FiO_2_ Ratio, Esophageal Pressure Swing, modified Pressure-Time Product and Work of Breathing, respectively and the Percentage of Well-aerated lung tissue in supine position.** The scatter-plot represents individual patient measurement-pairs, the black line displays the fitted linear regression, and the shaded gray area depicts its 95% Confidence Interval. *ρ ‒ Pearson correlation coefficient, p ‒ P-value.*


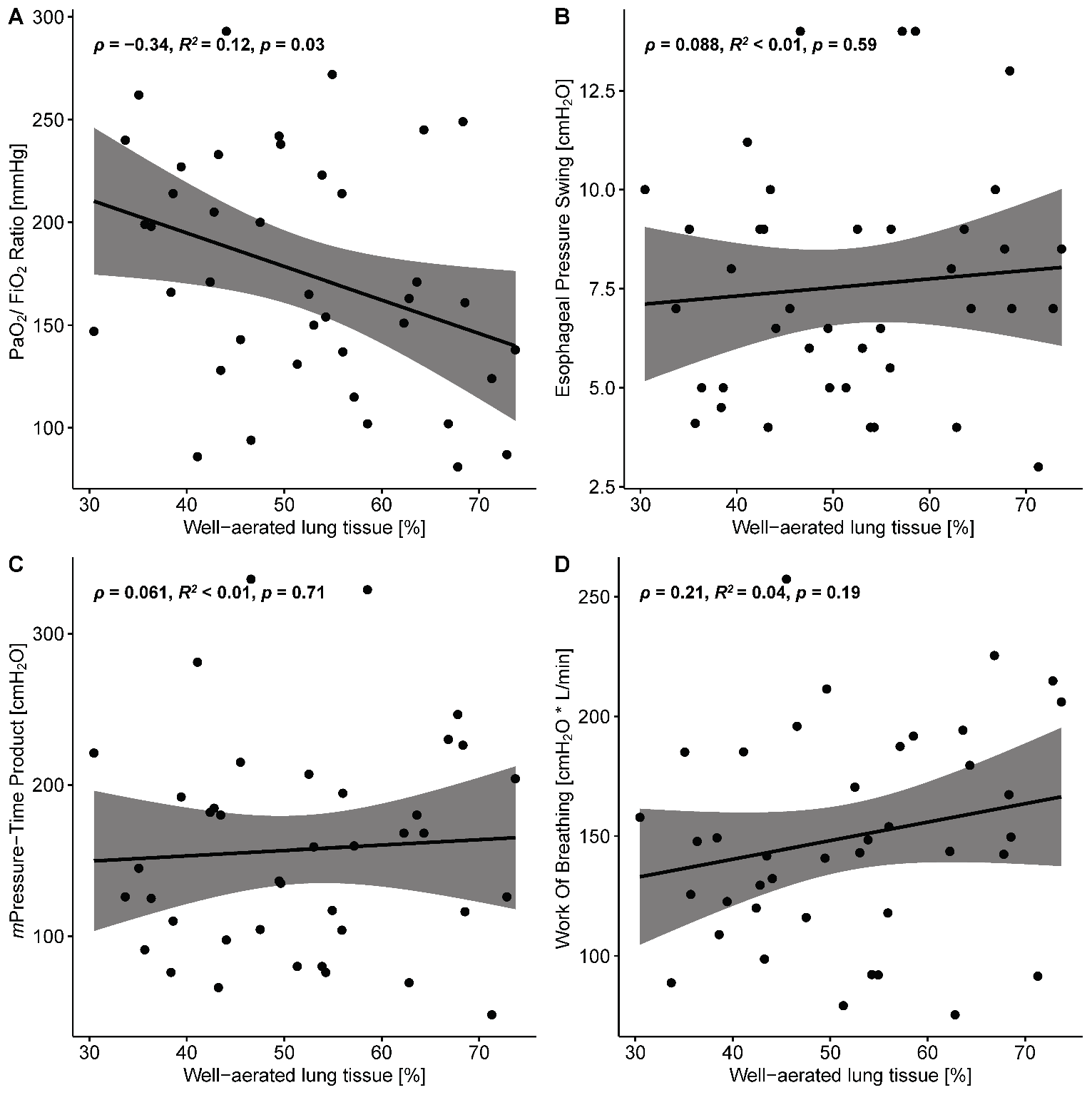


**
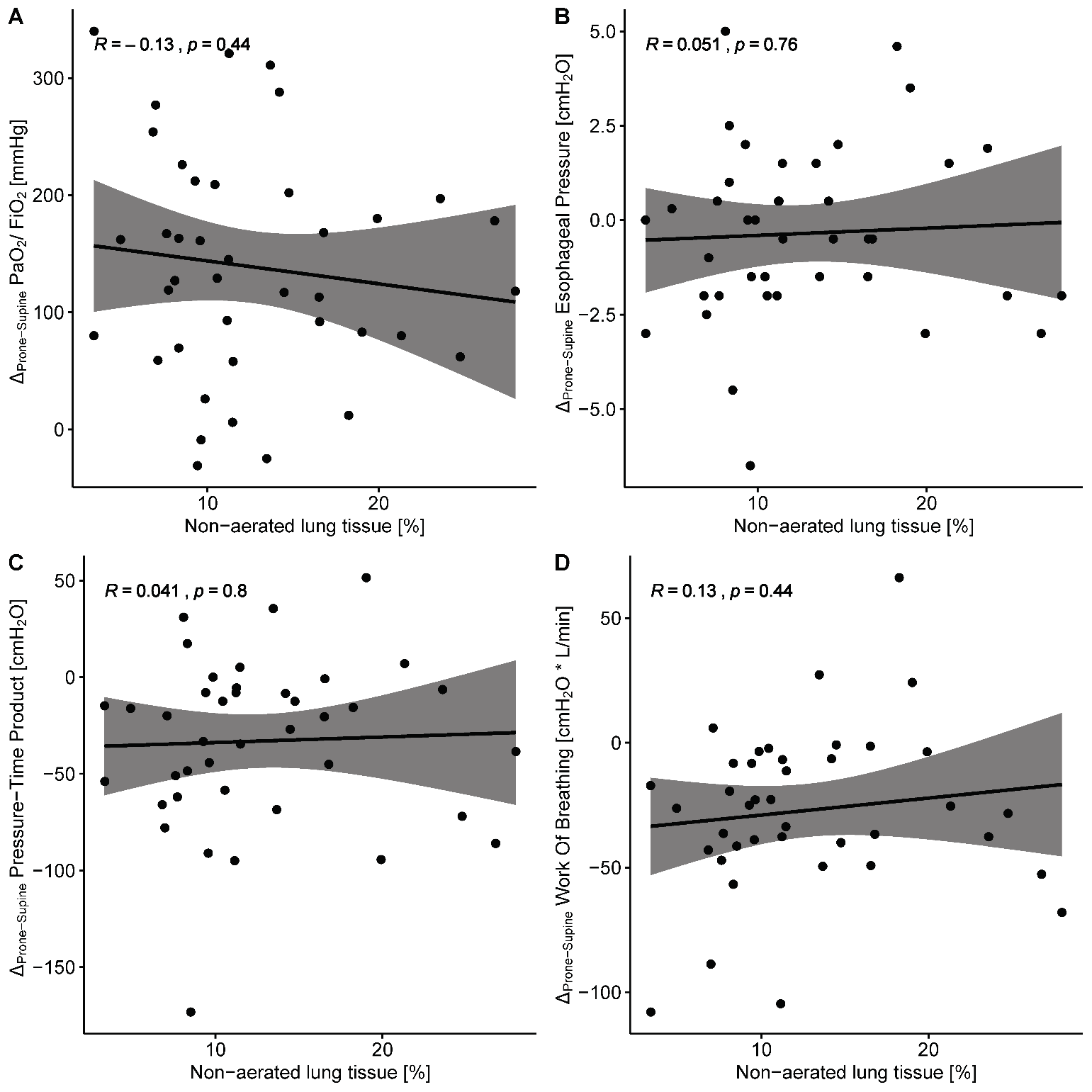
e-Figure 10. Correlation plots between the change in PaO_2_/FiO_2_ Ratio, Esophageal Pressure Swings, modified Pressure-Time Product and Work of Breathing from supine to prone position and the Percentage of Non-aerated lung tissue in supine position.** The scatter-plot represents individual patient measurement-pairs, the black line displays the fitted linear regression, and the shaded gray area depicts its 95% Confidence Interval. *ρ ‒ Pearson correlation coefficient, p ‒ P-value.*

**
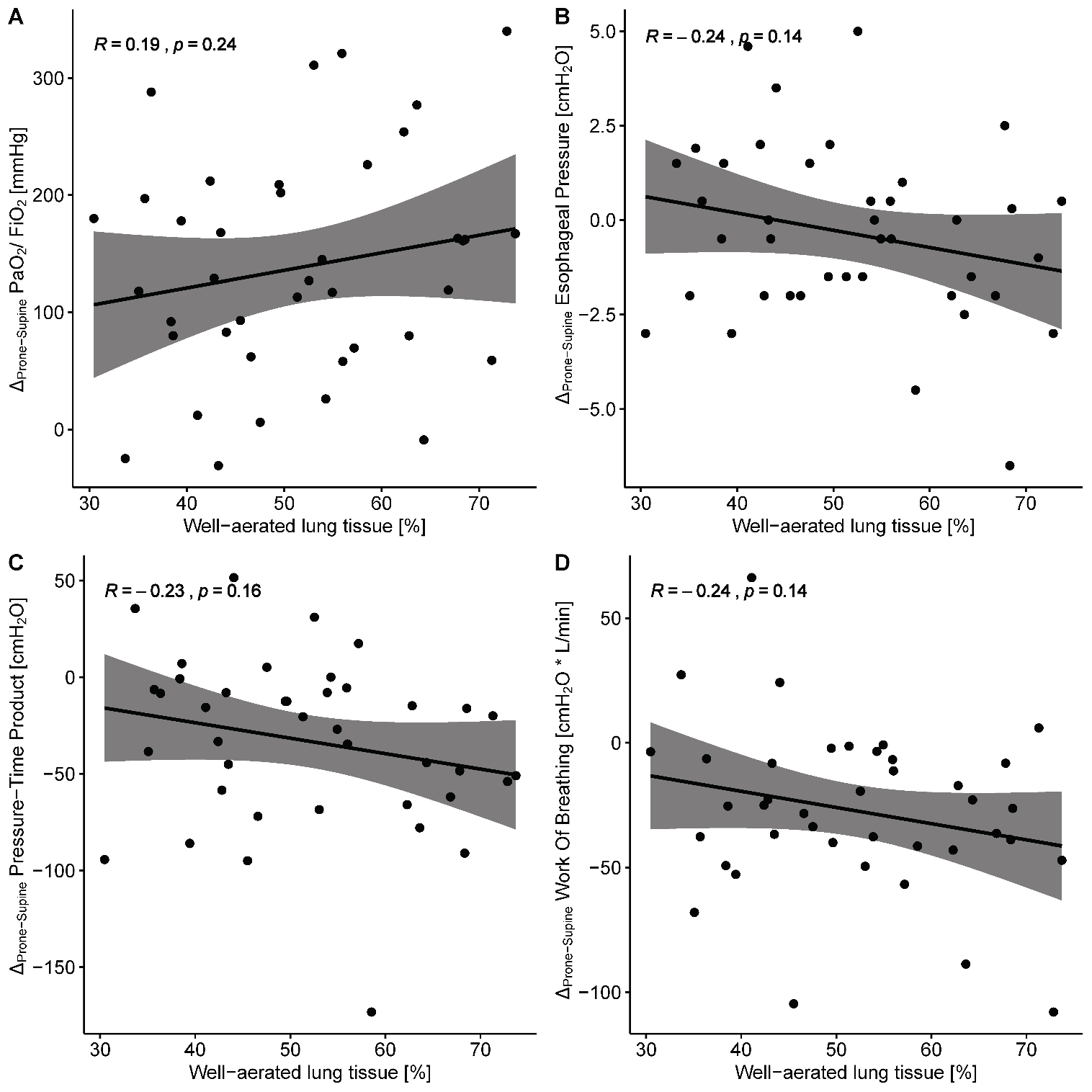
e-Figure 11. Correlation plots between the change in PaO_2_/ FiO_2_ Ratio, Esophageal Pressure Swings, modified Pressure-Time Product and Work of Breathing from supine to prone position and the Percentage of Well-aerated lung tissue in supine position.** The scatter-plot represents individual patient measurement-pairs, the black line displays the fitted linear regression, and the shaded gray area depicts its 95% Confidence Interval. *ρ ‒ Pearson correlation coefficient, p ‒ P-value***.**

**e-Figure 12. Correlation plots between the change in PaO_2_/FiO_2_ Ratio, Esophageal Pressure Swings, modified Pressure-Time Product and Work of Breathing from supine to prone position and the Percentage of Non-aerated Ventral lung tissue (A-D) and Non-aerated Dorsal lung tissue (E-H) in supine position.** The scatter-plot represents individual patient measurement-pairs, the black line isplays the fitted linear regression, and the shaded gray area depicts its 95% Confidence Interval. *ρ ‒ Pearson correlation coefficient, p ‒ P-value.*

**
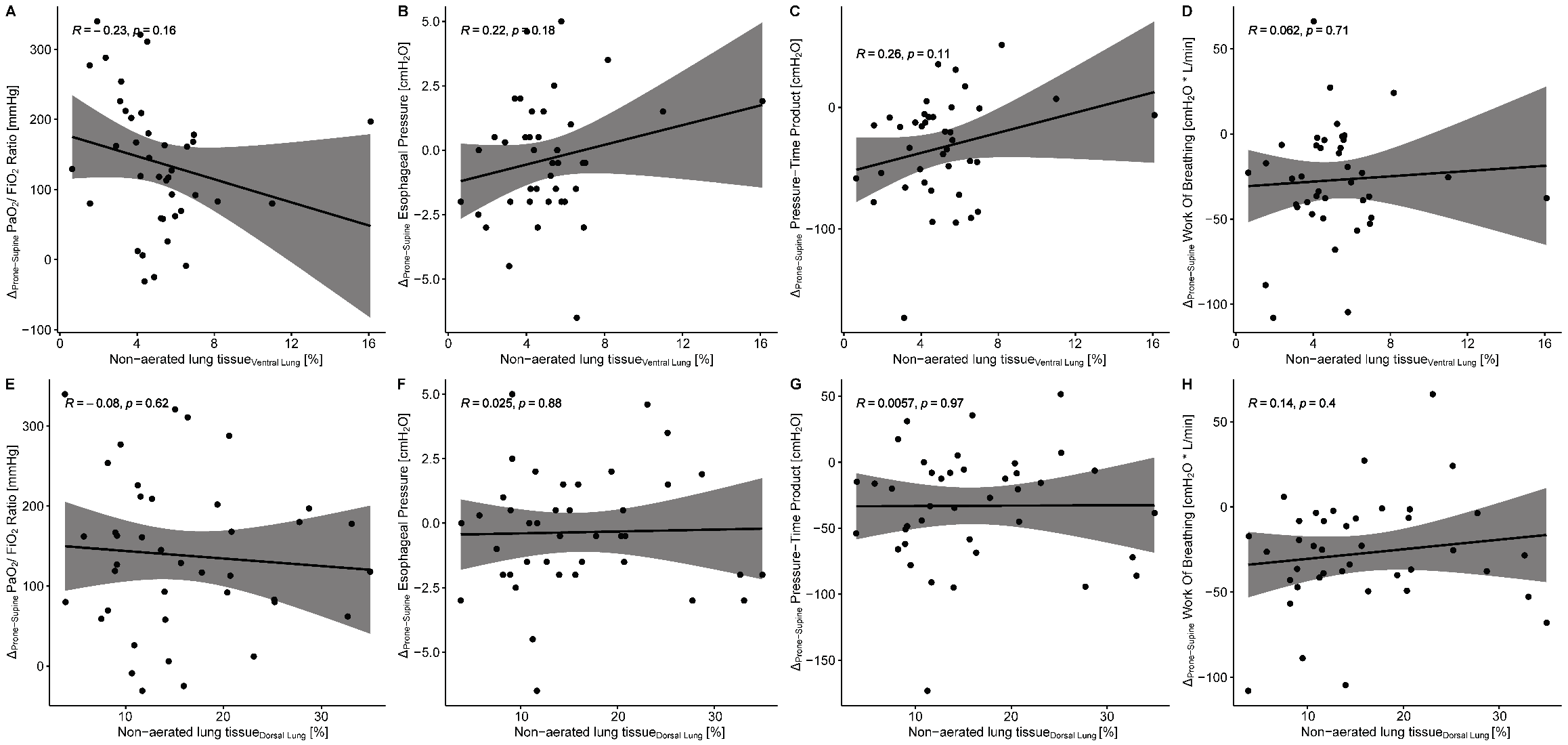
**

**e-Figure 13. Correlation plots between the change in PaO_2_/ FiO_2_ Ratio, Esophageal Pressure Swings, modified Pressure-Time Product and Work of Breathing from supine to prone position and the Percentage of Well-aerated Ventral lung tissue (A-D) and Well-aerated Dorsal lung tissue (E-H) in supine position.** The scatter-plot represents individual patient measurement-pairs, the black line displays the fitted linear regression, and the shaded gray area depicts its 95% Confidence Interval. *ρ ‒ Pearson correlation coefficient, p ‒ P-value***.**

**
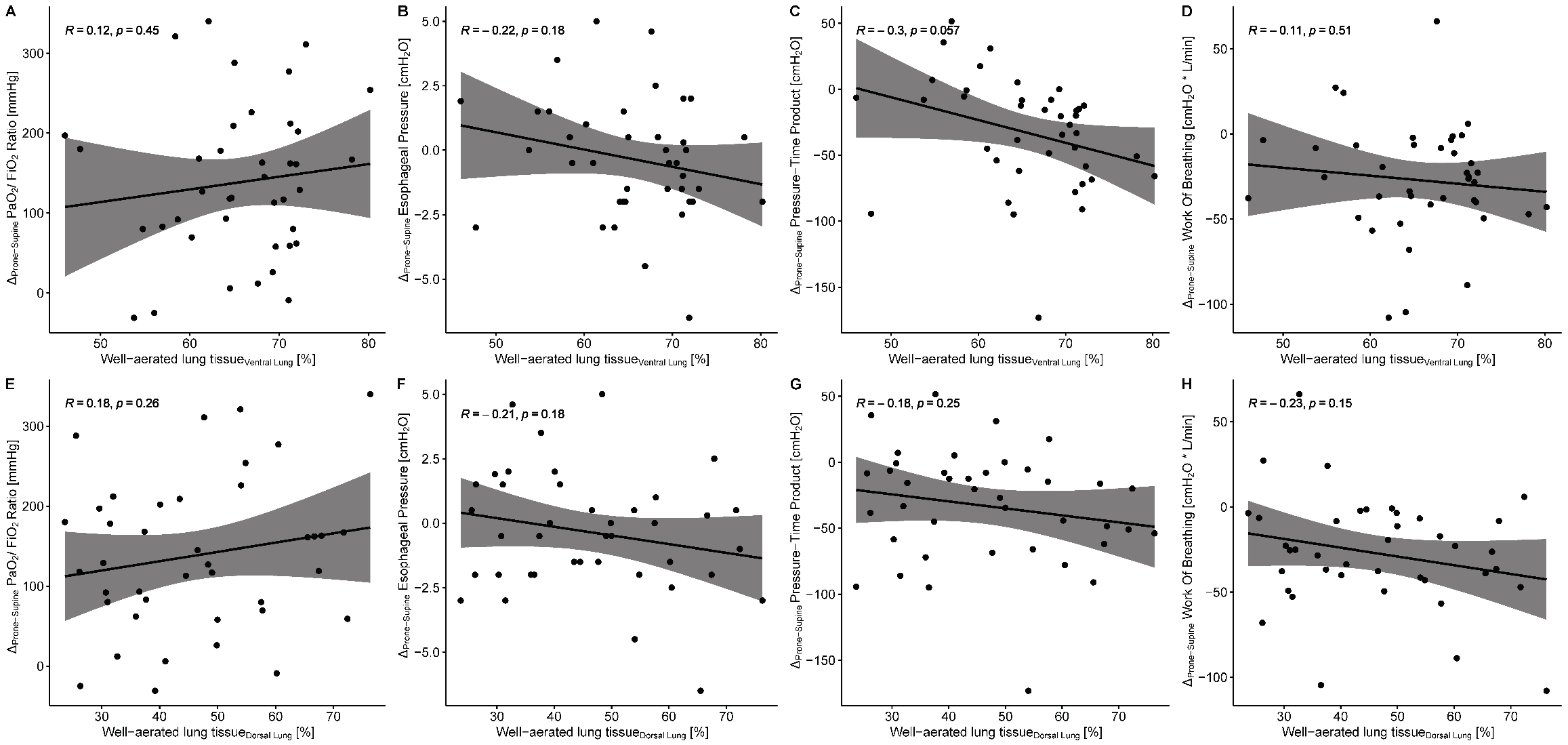
**


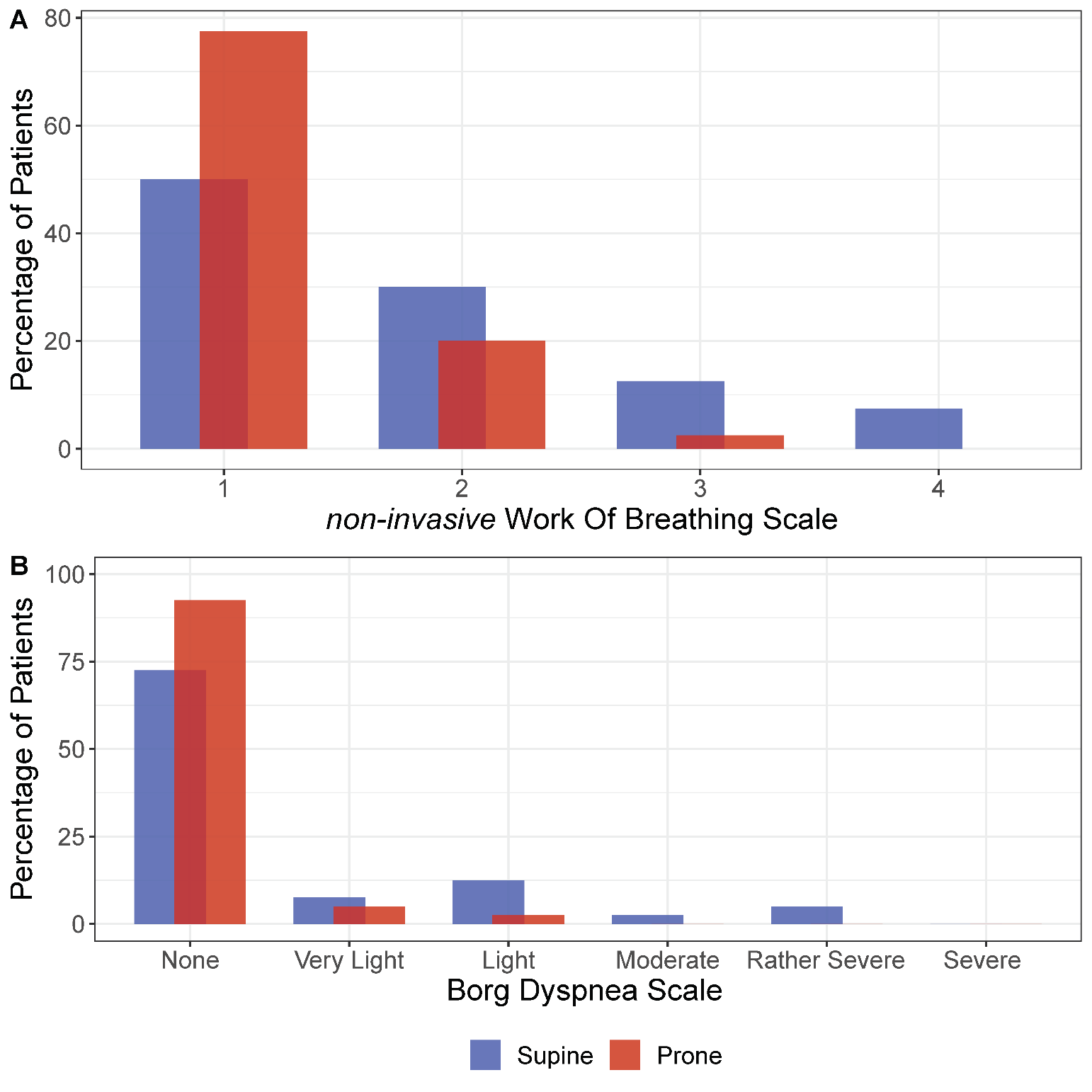
**e-Figure 14. Change in distribution of the non-invasive Work of Breathing Scale and the Borg Dyspnea Scale from supine to prone position.**

**e-Figure 15. Correlation plots between the Borg Dyspnea Scale and the modified Pressure-Time Product and Work of Breathing, respectively in supine position.** The scatter-plot represents individual patient measurement-pairs, the black line displays the fitted linear regression, and the shaded gray area depicts its 95% Confidence Interval. *τ ‒ Kendall correlation coefficient, p ‒ P-value.*


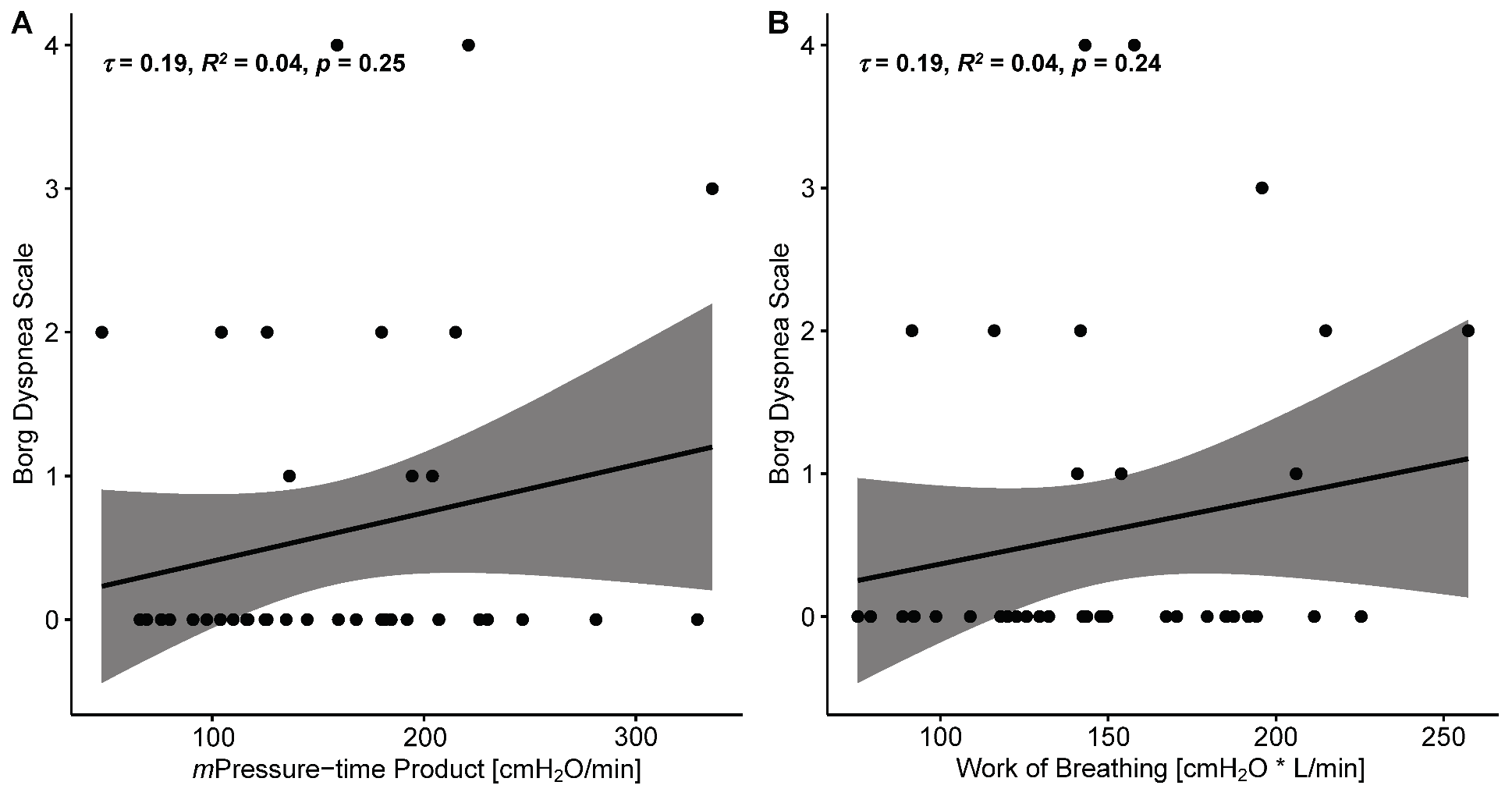


|  | PaO_2_/ FiO_2_ > 150 mmHg | | | | PaO_2_/ FiO_2_ ≤ 150 mmHg | | | |  |
| --- | --- | --- | --- | --- | --- | --- | --- | --- | --- |
|  | Supine  N = 25 | Prone  N = 25 | p | Mean Difference^†^ [95% CI] | Supine  N = 15 | Prone  N = 15 | p | Mean Difference^†^ [95% CI] | p* |
| Respiratory Rate, bpm | 20 [17, 22] | 17 [15, 19] | <0.001 | 3 [1, 4] | 23 [18, 25] | 18 [17, 20] | <0.001 | 4 [2, 6] | 0.171 |
| Tidal Volume, ml | 440 [380, 520] | 407 [374, 462] | 0.06 | 27 [-1, 55] | 400 [344, 477] | 435 [406, 496] | 0.692 | -14 [-90, 61] | 0.213 |
| Minute Ventilation, l/min | 9.0 [7.2, 10.6] | 7.1 [6.4, 8.2] | <0.001 | 1.7 [1.0, 2.4] | 8.2 [8.0, 10.9] | 7.8 [7.6, 9.4] | 0.119 | 0.8 [-0.2, 1.7] | 0.097 |
| PaO_2_/ FiO_2_ Ratio, mmHg | 214 [171, 240] | 376 [258, 405] | <0.001 | -136 [-176, -96] | 124 [98, 138] | 244 [190, 316] | <0.001 | -142 [-194, -91] | 0.832 |
| Estimated Dead-Space Fraction, % | 42 [36, 48] | 33 [24, 39] | 0.002 | 9 [4, 14] | 38 [30, 52] | 32 [22, 38] | 0.049 | 9 [0, 18] | 0.825 |
| Esophageal pressure swing, cmH_2_O | -7 [-8, -5] | -6 [-7, -5] | 0.912 | 0 [-1, 1] | -9 [-11, -7] | -9 [-10, -5] | 0.125 | -1 [-2, 0] | 0.253 |
| Dynamic Transpulmonary Pressure, cmH_2_O | 15 [14, 17] | 16 [14, 17] | 0.912 | 0 [-1, 1] | 19 [17, 21] | 18 [15, 19] | 0.169 | 1 [0, 2] | 0.293 |
| Modified Pressure-Time Product, cmH_2_O/min | 125 [98, 168] | 106 [90, 124] | 0.013 | 19 [4, 34] | 204 [159, 238] | 153 [105, 173] | <0.001 | 56 [31, 80] | 0.007 |
| Work of Breathing, cmH_2_O * L /min | 132 [116, 150] | 107 [90, 128] | <0.001 | 25 [14, 36] | 186 [143, 201] | 143 [106, 157] | 0.015 | 31 [7, 55] | 0.602 |
| Non-aerated lung tissue, % | 11 [9, 15] |  |  |  | 11 [8, 17] |  |  |  | 0.591 |
| Well aerated lung tissue, % | 50 [39, 56] |  |  |  | 56 [46, 67] |  |  |  | 0.024 |

**e-Table 3. Subgroup Analysis for PaO_2_/ FiO_2_ >150 mmHg and ≤150 mmHg.**

*Definition of abbreviations*: CI = confidence interval; bpm= breaths per minute. Data are presented as median [interquartile range] for continuous values unless otherwise specified. ^†^Supine Position taken as reference. *p-value for the difference of effect between Supine and Position, between Groups (PaO_2_/ FiO_2_ >150 mmHg and ≤150 mmHg), calculated with a mixed-effects model considering Group and Intervention (Supine/ Prone) as fixed effects with interaction term and Patient as a random effect.

|  | Esophageal Pressure Swing > 7 cmH_2_O | | | | Esophageal Pressure Swing ≤ 7 cmH_2_O | | | |  |
| --- | --- | --- | --- | --- | --- | --- | --- | --- | --- |
|  | Supine  N = 18 | Prone  N = 18 | p | Mean Difference^†^ [95% CI] | Supine  N = 22 | Prone  N = 22 | p | Mean Difference^†^ [95% CI] | p* |
| Respiratory Rate, bpm | 22 [20, 24] | 17 [16, 19] | <0.001 | 4 [2, 6] | 18 [17, 22] | 17 [15, 19] | 0.002 | 3 [1, 4] | 0.177 |
| Tidal Volume, ml | 382 [350, 461] | 410 [376, 466] | 0.872 | -4 [-59, 50] | 480 [396, 522] | 427 [380, 496] | 0.233 | 24 [-17, 64] | 0.380 |
| Minute Ventilation, l/min | 8.2 [8.0, 9.0] | 7.5 [6.6, 8.1] | 0.006 | 1.2 [0.4, 2.0] | 9.3 [6.8, 10.6] | 7.8 [6.6, 8.9] | 0.001 | 1.5 [0.7, 2.3] | 0.615 |
| PaO_2_/ FiO_2_ Ratio, mmHg | 143 [105, 171] | 316 [227, 382] | <0.001 | -149 [-184, -114] | 200 [156, 237] | 309 [236, 419] | 0.001 | -130 [-180, -80] | 0.538 |
| Estimated Dead-Space Fraction, % | 41 [35, 46] | 32 [19, 38] | 0.010 | 10 [3, 16] | 44 [35, 51] | 33 [24, 45] | 0.008 | 8 [2, 15] | 0.725 |
| Esophageal pressure swing, cmH_2_O | -9 [-11, -9] | -9 [-11, -7] | 0.221 | -1 [-2, 1] | -5 [-7, -4] | -5 [-6, -4] | 0.925 | 0 [-1, 1] | 0.217 |
| Dynamic Transpulmonary Pressure, cmH_2_O | 19 [18, 21] | 18.2 [16.6, 19.4] | 0.257 | 1 [-1, 2] | 14 [13, 16] | 14 [13, 16] | 0.925 | 0 [-1, 1] | 0.250 |
| Modified Pressure-Time Product, cmH_2_O/min | 199 [180, 229] | 151 [126, 175] | <0.001 | 56 [33, 78] | 107 [80, 126] | 94 [73, 119] | 0.038 | 15 [1, 29] | 0.002 |
| Work of Breathing, cmH_2_O * L /min | 169 [143, 191] | 132 [106, 15] | <0.001 | 31 [15, 47] | 129 [94, 149] | 103 [89, 135] | 0.003 | 24 [9, 39] | 0.488 |
| Non-aerated lung tissue, % | 9 [8, 18] |  |  |  | 11 [10, 15] |  |  |  | 0.472 |
| Well aerated lung tissue, % | 54 [43, 63] |  |  |  | 51 [44, 56] |  |  |  | 0.574 |

**e-Table 4. Subgroup Analysis for Esophageal Pressure Swing >7 cmH_2_O and ≤7 cmH_2_O.**

*Definition of abbreviations*: CI = confidence interval; bpm= breaths per minute. Data are presented as median [interquartile range] for continuous values unless otherwise specified. ^†^Supine Position taken as reference. *p-value for the difference of effect between Supine and Position, between Groups (Esophageal Pressure Swing >7 cmH_2_O and ≤7 cmH_2_O), calculated with a mixed-effects model considering Group and Intervention (Supine/ Prone) as fixed effects with interaction term and Patient as a random effect.

**e-Table 5. Subgroup Analysis for Dynamic Lung Compliance >64 ml/cmH_2_O and ≤64 ml/cmH_2_O.**

|  | Dynamic Lung Compliance > 64^§^ ml/cmH_2_O | | | | Dynamic Lung Compliance ≤ 64^§^ ml/cmH_2_O | | | |  |
| --- | --- | --- | --- | --- | --- | --- | --- | --- | --- |
|  | Supine  N = 32 | Prone  N = 32 | p | Mean Difference^†^ [95% CI] | Supine  N = 20 | Prone  N = 20 | p | Mean Difference^†^ [95% CI] | p* |
| Respiratory Rate, bpm | 18 [17, 22] | 17 [15, 19] | 0.002 | 3 [1, 4] | 22 [20, 24] | 18 [16, 19] | <0.001 | 4 [2, 5] | 0.322 |
| Tidal Volume, ml | 490 [416, 530] | 438 [386, 506] | 0.08 | 40 [-5, 85] | 378 [340, 442] | 408 [373, 454] | 0.436 | -17 [-63, 28] | 0.068 |
| Minute Ventilation, l/min | 7.8 [6.8, 8.6] | 9.3 [7.0, 10.6] | <0.001 | 1.7 [0.8, 2.6] | 8.1 [7.8, 9.4] | 7.5 [6.4, 8.4] | 0.012 | 1.0 [0.2, 1.7] | 0.200 |
| PaO_2_/ FiO_2_ Ratio, mmHg | 198 [153, 226] | 346 [241, 430] | <0.001 | -145 [-195, -94] | 149 [112, 210] | 300 [220, 384] | <0.001 | -132 [172, -93] | 0.686 |
| Estimated Dead-Space Fraction, % | 44 [37, 52] | 32 [24, 42] | 0.001 | 11 [5, 18] | 37 [30, 47] | 34 [22, 39] | 0.055 | 6 [0, 12] | 0.264 |
| Esophageal pressure swing, cmH_2_O | -5 [-6, -4] | -5 [-7, -4] | 0.914 | 0 [-1, 1] | -9 [-10, -8] | -8 [-11, -6] | 0.270 | -1 [-2, 1] | 0.360 |
| Dynamic Transpulmonary Pressure, cmH_2_O | 14 [13, 15] | 14 [12, 16] | 0.978 | 0 [-1, 1] | 19 [17, 20] | 18 [16, 19] | 0.310 | 1 [-1, 2] | 0.399 |
| Modified Pressure-Time Product, cmH_2_O/min | 104 [79, 128] | 94 [72, 117] | 0.026 | 16 [2, 30] | 193 [177, 227] | 151 [125, 170] | <0.001 | 50 [28, 72] | 0.011 |
| Work of Breathing, cmH_2_O * L /min | 137 [106, 149] | 103 [89, 127] | <0.001 | 30 [14, 46] | 163 [139, 189] | 132 [105, 155] | 0.003 | 25 [9, 40] | 0.621 |
| Non-aerated lung tissue, % | 11 [10, 17] |  |  |  | 10 [8, 15] |  |  |  | 0.612 |
| Well aerated lung tissue, % | 50 [42, 55] |  |  |  | 55 [43, 64] |  |  |  | 0.410 |

*Definition of abbreviations*: CI = confidence interval; bpm= breaths per minute. Data are presented as median [interquartile range] for continuous values unless otherwise specified. ^†^Supine Position taken as reference. ^§^The cut-off 64 ml/cmH_2_O was chosen as it represents the median of the dynamic lung compliance for this cohort. *p-value for the difference of effect between Supine and Position, between Groups (Dynamic Lung Compliance >64 ml/cmH_2_O and ≤64 ml/cmH_2_O), calculated with a mixed-effects model considering Group and Intervention (Supine/ Prone) as fixed effects with interaction term and Patient as a random effect.

|  | Subjective Sensation of Dyspnea | | | | No Subjective Sensation of Dyspnea | | | |  |
| --- | --- | --- | --- | --- | --- | --- | --- | --- | --- |
|  | Supine  N = 11 | Prone  N = 11 | p | Mean Difference^†^ [95% CI] | Supine  N = 29 | Prone  N = 29 | p | Mean Difference^†^ [95% CI] | p* |
| Respiratory Rate, bpm | 22 [18, 24] | 18 [16, 21] | <0.001 | 3 [2, 5] | 20 [17, 23] | 17 [15, 19] | <0.001 | 3 [2, 5] | 0.906 |
| Tidal Volume, ml | 450 [388, 486] | 424 [399, 470] | 0.669 | 16 [-63, 94] | 418 [348, 520] | 409 [374, 477] | 0.586 | 10 [-26, 46] | 0.870 |
| Minute Ventilation, l/min | 8.1 [8.0, 10.9] | 8.2 [7.8, 9.4] | 0.123 | 1.1 [-0.3, 2.5] | 8.7 [7.2, 10.6] | 7.2 [6.4, 8.2] | <0.001 | 1.5 [0.8, 2.1] | 0.528 |
| PaO_2_/ FiO_2_ Ratio, mmHg | 138 [126, 149] | 296 [201, 377] | <0.001 | -150 [-222, -78] | 198 [154, 233] | 328 [243, 396] | <0.001 | -134 [-169, -99] | 0.636 |
| Estimated Dead-Space Fraction, % | 48 [36, 54] | 38 [28, 45] | 0.09 | 9 [-2, 19] | 41 [33, 48] | 32 [22, 38] | <0.001 | 9 [4, 14] | 0.881 |
| Esophageal pressure swing, cmH_2_O | -7 [-10, -6] | -7 [-9, -5] | 0.02 | -1 [-2, 0] | -7 [-9, -5] | -7 [-9, -6] | 0.883 | 0 [-1, 1] | 0.172 |
| Dynamic Transpulmonary Pressure, cmH_2_O | 17 [14, 19] | 15 [13, 18] | 0.02 | 1 [0, 2] | 17 [15, 19] | 17 [15, 18] | 0.931 | 0 [-1, 1] | 0.169 |
| Modified Pressure-Time Product, cmH_2_O/min | 180 [131, 209] | 124 [100, 144] | <0.001 | 49 [28, 71] | 135 [98, 185] | 117 [90, 149] | 0.003 | 27 [10, 44] | 0.142 |
| Work of Breathing, cmH_2_O * L /min | 154 [141, 201] | 139 [101, 154] | 0.008 | 38 [12, 64] | 144 [118, 180] | 111 [91, 141] | <0.001 | 23 [11, 35] | 0.201 |
| Non-aerated lung tissue, % | 12 [9, 15] |  |  |  | 11 [8, 17] |  |  |  | 0.796 |
| Well aerated lung tissue, % | 50 [46, 64] |  |  |  | 53 [41, 62] |  |  |  | 0.432 |

**e-Table 6. Subgroup Analysis for Subjective Sensation Dyspnea versus None.**

*Definition of abbreviations*: CI = confidence interval; bpm= breaths per minute. Data are presented as median [interquartile range] for continuous values unless otherwise specified. ^†^Supine Position taken as reference. *p-value for the difference of effect between Supine and Position, between Groups (Subjective Sensation Dyspnea versus None), calculated with a mixed-effects model considering Group and Intervention (Supine/ Prone) as fixed effects with interaction term and Patient as a random effect.

**REFERENCES**

E1. Sinha P, Calfee CS, Beitler JR, et al: Physiologic Analysis and Clinical Performance of the Ventilatory Ratio in Acute Respiratory Distress Syndrome. *Am J Respir Crit Care Med* 2019; 199:333-341.

E2. Morales-Quinteros L, Schultz MJ, Bringué J, et al: Estimated dead space fraction and the ventilatory ratio are associated with mortality in early ARDS. *Ann Intensive Care* 2019; 9:128.
